# Identification of a Proline-Kinked Amphipathic α-Helix Downstream from the Methyltransferase Domain of a Potexvirus Replicase and Its Role in Virus Replication and Perinuclear Complex Formation

**DOI:** 10.1128/JVI.01906-20

**Published:** 2021-09-27

**Authors:** Ken Komatsu, Nobumitsu Sasaki, Tetsuya Yoshida, Katsuhiro Suzuki, Yuki Masujima, Masayoshi Hashimoto, Satoru Watanabe, Naoya Tochio, Takanori Kigawa, Yasuyuki Yamaji, Kenro Oshima, Shigetou Namba, Richard S. Nelson, Tsutomu Arie

**Affiliations:** a Laboratory of Plant Pathology, Graduate School of Agriculture, Tokyo University of Agriculture and Technology (TUAT), Fuchu, Japan; b Institute of Global Innovation Research (GIR), Tokyo University of Agriculture and Technology (TUAT), Fuchu, Japan; c Gene Research Center, Tokyo University of Agriculture and Technology (TUAT), Fuchu, Japan; d Laboratory of Plant Pathology, Graduate School of Agricultural and Life Sciences, The University of Tokyo, Tokyo, Japan; e Laboratory for Cellular Structural Biology, RIKEN Center for Biosystems Dynamics Research, Yokohama, Japan; f Faculty of Bioscience, Department of Clinical Plant Science, Hosei University, Koganei, Japan; g Department of Entomology and Plant Pathology, Oklahoma State University, Stillwater, Oklahoma, USA; University of Maryland, College Park

**Keywords:** NMR, amphipathic α-helix, endoplasmic reticulum, methyltransferase, *Plantago asiatica* mosaic virus, potexvirus, *Tymovirales*, virus replication

## Abstract

Characterized positive-strand RNA viruses replicate in association with intracellular membranes. Regarding viruses in the genus *Potexvirus*, the mechanism by which their RNA-dependent RNA polymerase (replicase) associates with membranes is understudied. Here, by membrane flotation analyses of the replicase of Plantago asiatica mosaic potexvirus (PlAMV), we identified a region in the methyltransferase (MET) domain as a membrane association determinant. An amphipathic α-helix was predicted downstream from the core region of the MET domain, and hydrophobic amino acid residues were conserved in the helical sequences in replicases of other potexviruses. Nuclear magnetic resonance (NMR) analysis confirmed the amphipathic α-helical configuration and unveiled a kink caused by a highly conserved proline residue in the α-helix. Substitution of this proline residue and other hydrophobic and charged residues in the amphipathic α-helix abolished PlAMV replication. Ectopic expression of a green fluorescent protein (GFP) fusion with the entire MET domain resulted in the formation of a large perinuclear complex, where virus replicase and RNA colocated during virus infection. Except for the proline substitution, the amino acid substitutions in the α-helix that abolished virus replication also prevented the formation of the large perinuclear complex by the respective GFP-MET fusion. Small intracellular punctate structures were observed for all GFP-MET fusions, and *in vitro* high-molecular-weight complexes were formed by both replication-competent and -incompetent viral replicons and thus were not sufficient for replication competence. We discuss the roles of the potexvirus-specific, proline-kinked amphipathic helical structure in virus replication and intracellular large complex and punctate structure formation.

**IMPORTANCE** RNA viruses characteristically associate with intracellular membranes during replication. Although virus replicases are assumed to possess membrane-targeting properties, their membrane association domains generally remain unidentified or poorly characterized. Here, we identified a proline-kinked amphipathic α-helix structure downstream from the methyltransferase core domain of PlAMV replicase as a membrane association determinant. This helical sequence, which includes the proline residue, was conserved among potexviruses and related viruses in the order *Tymovirales*. Substitution of the proline residue, but not the other residues necessary for replication, allowed formation of a large perinuclear complex within cells resembling those formed by PlAMV replicase and RNA during virus replication. Our results demonstrate the role of the amphipathic α-helix in PlAMV replicase in a perinuclear complex formation and virus replication and that perinuclear complex formation by the replicase alone will not necessarily indicate successful virus replication.

## INTRODUCTION

Positive-strand RNA viruses are a large group of viruses, including many clinically important human, animal, and plant pathogens. Replication of characterized positive-strand RNA viruses occurs in close association with intracellular membranes, such as those in endoplasmic reticulum (ER), the Golgi apparatus, mitochondria, and chloroplasts. Replication is accompanied by dynamic membrane remodeling that often induces the formation of large inclusion structures and spherular vesicles, both of which are sometimes recognized as virus replication complexes (VRCs) (1). Virus-induced membrane remodeling is thought to lead to efficient replication by recruiting virus and host components for VRC formation, as well as by protecting viral RNA from host cellular nucleases (2–6).

Remodeling of host intracellular membranes during VRC formation is proposed to be mediated by virus-encoded replicase and auxiliary replication proteins, all of which possess membrane-targeting properties (1, 7). Some of these proteins have a transmembrane domain that contributes to membrane targeting (8, 9). On the other hand, an increasing number of reports have demonstrated that replication-related proteins contain an amphipathic α-helix structure involved in membrane association and remodeling and subsequent virus replication (10–14). However, due to the lack of conserved amino acid motifs in the known amphipathic helices, it is difficult to identify an amphipathic α-helix structure from a primary amino acid sequence that, with certainty, is involved in membrane association and virus replication. Thus, amphipathic α-helix structures that function as a membrane association domain of a replicase and function in virus replication remain unidentified in a variety of viruses.

To date, three-dimensional (3D) structures of amphipathic α-helices in several viral membrane-associated proteins, including hepatitis C virus (HCV) NS4B and NS5A proteins and brome mosaic virus (BMV) 1a protein, have been resolved by nuclear magnetic resonance (NMR) (11, 13, 15–17). The overall linear structures of these resolved amphipathic α-helices are essentially similar and likely to bind to the lipid bilayer. Amino acid residues located in the hydrophobic side of the amphipathic helix are relatively conserved among closely related viruses and have been shown to play a key role in membrane targeting (11, 17). On the opposite side of the amphipathic α-helix is a polar cytosolic face with charged and basic residues which may mediate electrostatic interactions with the negatively charged phospholipids in the intracellular membranes as well as self-interactions and interactions with other host proteins (18, 19). Indeed, mutations of hydrophobic or hydrophilic/polar amphipathic α-helix amino acid residues in viral replicases have been shown to decrease protein stability or the efficiency of viral replication (11, 13). These combined findings indicate that the tertiary structure and membrane association properties of an amphipathic α-helix of a virus replicase are important for the very early phase in virus replication.

**FIG 1:**
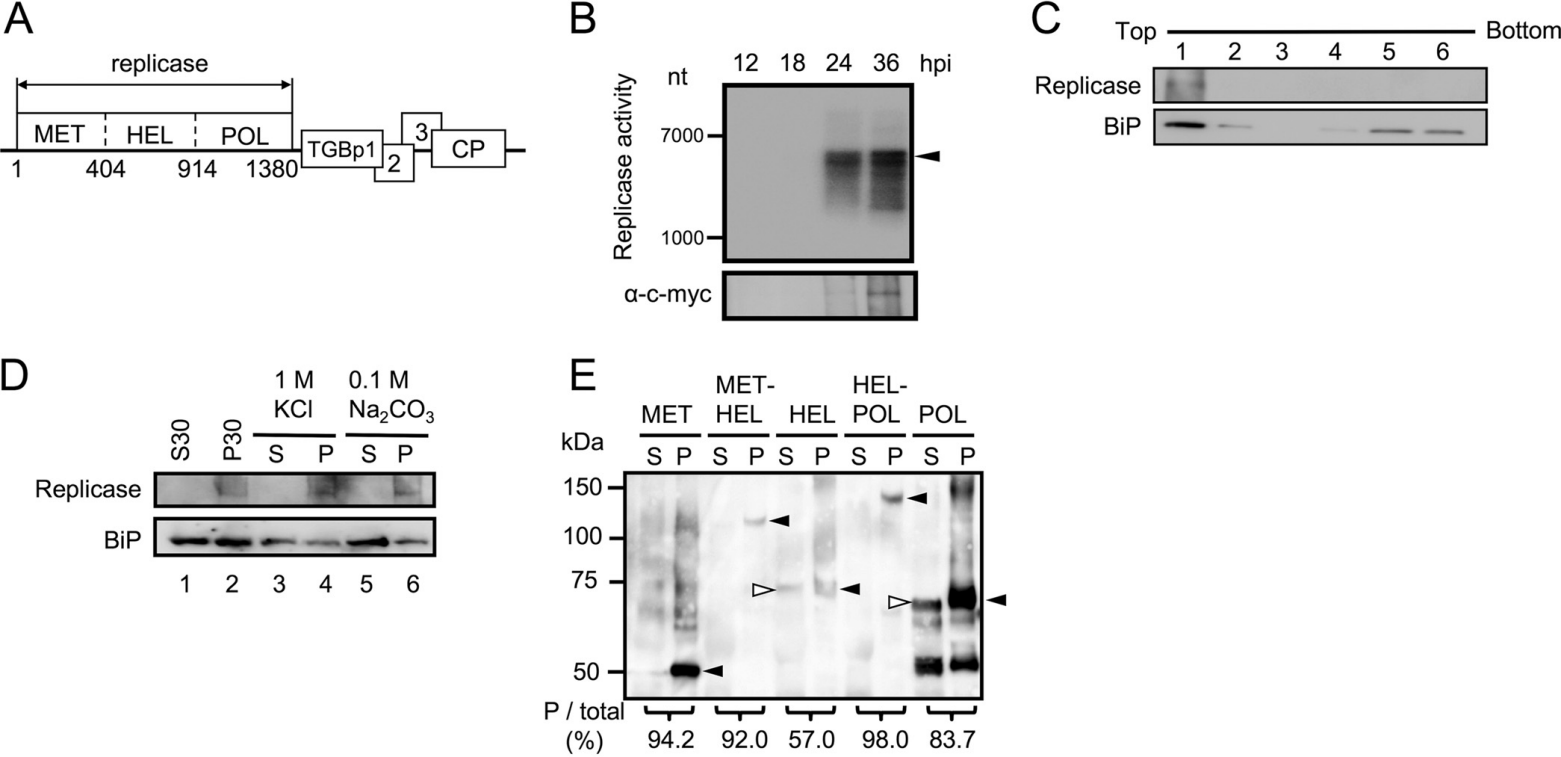
Replicase of PlAMV is tightly associated with ER membranes by its N-terminal MET domain. (A) Schematic representation of the genomic organization of PlAMV. Rectangles represent open reading frames (ORFs). The three domains of the viral replicase, methyltransferase (MET), helicase (HEL), and polymerase (POL), are shown. Numbers below the replicase ORF refer to amino acid positions delimiting replicase domains within the Li1 isolate. (B) *In vitro* replicase activity by the membrane-associated P30 fraction from tissue infected with 53U-RdRp-myc, a minimum replicon of PlAMV. Reactions were carried out using the P30 fractions extracted from N. benthamiana leaves at 12, 18, 24, and 36 h postinoculation with 53U-RdRp-myc. (Upper panel) ^32^P-labeled RNA products synthesized by PlAMV replicase were dissolved in 8 M urea and subjected to 2.4% polyacrylamide gel electrophoresis, followed by autoradiography. The arrowhead indicates bands corresponding to the full-length genomic RNA of 53U-RdRp-myc. (Lower panel) Immunoblot analysis for the presence of PlAMV replicase in the each P30 fraction, detected by anti-c-*myc* antibody. (C) Membrane flotation assay and immunoblot analysis of P30 samples extracted from leaves expressing c-*myc*-tagged PlAMV replicase. Immunoblotting was performed using anti-c-*myc* (band indicated as Replicase) and anti-BiP (band indicated as BiP) antibodies. BiP is a resident ER protein. (D) Biochemical analysis of the full-length, c-*myc*-tagged PlAMV replicase expressed from the estradiol-inducible expression vector pER8. At 2.5 days after estradiol treatment, protein was extracted and fractionated into S30 and P30 fractions (lanes 1 and 2). The P30 fraction was treated with the reagents indicated above the panel and subjected to centrifugation at 30,000 × *g* to separate the soluble (S) and pellet (P) fractions (lanes 3 to 6). Immunoblotting was performed using anti-c-*myc* and anti-BiP antibodies. (E) Immunoblot analysis showing subcellular localization of single or adjoining domains of PlAMV replicase. Each domain tagged with c-*myc* at the C terminus was expressed by agroinfiltration, followed by protein extraction, fractionation, and detection with anti-c-*myc* antibody. Black and white arrowheads indicate the detected domains of the expected molecular weights in the P30 and S30 fractions, respectively. Band intensities were quantified with ImageJ, and the percentages of total intensity in the P30 fraction are given at the bottom of the paired lanes for each domain treatment.

The genus *Potexvirus* is a group of filamentous positive-strand RNA viruses in the alphavirus supergroup, in the family *Alphaflexiviridae* of the order *Tymovirales*, and contains member viruses that cause severe damage to a variety of crop and ornamental plants (20). The potexvirus genome encodes at least five proteins: a replicase (RNA-dependent RNA polymerase [RdRp]) in open reading frame 1 (ORF1), three overlapping triple-gene block proteins (TGBs) involved in virus cell-to-cell movement in ORFs 2, 3, and 4, and a coat protein in ORF5 (20). The replicase contains a methyltransferase (MET) domain, a helicase (HEL) domain, and an RNA polymerase (POL) domain in order from the N terminus to C terminus (Fig. 1A). For potato virus X (PVX), the type species of the genus, the replicase is sufficient for successful virus replication in the absence of other viral proteins (21). Previous studies have indicated that, as is the case for other RNA viruses, infection with PVX is associated with host intracellular membranes: for example, an *in vitro* replication assay suggested that PVX replicates at the ER membranes (22). Through confocal and electron microscopy using membrane markers and immunodetection of the PVX replicase, the replicase was shown to be associated with membranes derived from the ER (23). Additionally, consistent with an older electron microscopic analysis, recent confocal laser scanning microscopy (CLSM)-based imaging analysis using fluorescent protein tags demonstrated that PVX-infected cells produce large inclusion bodies, or X-bodies, that contain ER-derived membranes (24–26). These results indicate that the replicase of PVX targets to and associates with ER membranes to form VRCs, similarly to the cases for other well-studied plant RNA viruses, including BMV (1). However, the mechanism by which the replicase associates with ER membranes and the requirement of this association for various steps within the replication cycle are understudied (23, 24, 27, 28).

Plantago asiatica mosaic virus (PlAMV) in the genus *Potexvirus* causes systemic necrosis in ornamental lilies (*Lilium* spp.) (29). PlAMV has been isolated from a wide variety of host plants, although members of the *Potexvirus* genus generally have narrow host ranges (30, 31). Our studies have shown that the replicase of PlAMV, which is sufficient for replication without other viral proteins, is a symptom determinant and is involved in induction of systemic necrosis in the model plant Nicotiana benthamiana (32, 33). We also determined that a plant jacalin-lectin resistance protein JAX1 inhibits PlAMV replication by targeting the replicase (34, 35). For the development of efficient virus control strategies, further understanding of the mechanism of PlAMV replication at the molecular level is required.

In the current study, through flotation assays and *in silico* structural prediction, we identified a membrane association domain in the MET of PlAMV replicase. This domain contained a putative α-helix amphipathic sequence. An amino acid alignment including members of related families further suggested that amphipathic α-helix structures are conserved among potexviruses and related RNA viruses in the order *Tymovirales*. An NMR-based three-dimensional structural analyses of a 28-amino-acid region in the membrane association domain of PlAMV replicase unveiled a novel proline-kinked amphipathic α-helix within this replicase and other members of the genus *Potexvirus*. Mutational analysis of amino acids within the α-helix structure indicated that those involved in the kink and the amphipathicity are crucial for negative-strand RNA synthesis of PlAMV. The need to conserve these amino acids to allow perinuclear complex formation by the MET domain and whether perinuclear complex formation correlated with virus replication were studied through cell biology methods.

## RESULTS

### Replicase of PlAMV tightly associates with host ER membranes.

We have recently shown by subcellular fractionation and immunoblot analyses that the replicase of PlAMV predominantly accumulated in the membrane fraction (P30) of N. benthamiana plants infected with the virus (33). A similar result was obtained by using 53U-RdRp-myc, a PlAMV replicon containing only the ORF for the replicase and the flanking 5′ and 3′ untranslated regions required for replication (33). Since the 53U-RdRp-myc does not contain ORFs encoding movement proteins or a coat protein, this result demonstrated that the enrichment of the replicase in P30 fraction is independent of other virus proteins. In this study, we first performed an *in vitro* replication assay using the P30 fraction and radiolabeled nucleotides. The P30 fraction from the PlAMV-infected N. benthamiana plants exhibited detectable *in vitro* replication activity at 24 h postinoculation (hpi) (Fig. 1B), similar to findings reported for PVX and bamboo mosaic potexvirus (BaMV) (22, 36). Labeled products were not degraded after treatment with S1 nuclease and require GTP and UTP, indicating the RNA is not end labeled (K. Komatsu, unpublished data). A membrane flotation analysis using a sucrose gradient showed that the replicase in the P30 fraction floated predominantly toward the top of the gradient, together with the ER luminal binding protein (BiP) (Fig. 1C). These results indicated that PlAMV replicase was associated with the ER, if not other intracellular membranes, and not aggregated in the P30 fraction. To further examine the membrane association of PlAMV replicase, the P30 fraction was prepared from plant leaves expressing c-*myc*-tagged PlAMV replicase and separated to solubilized (S) and nonsolubilized (P) fractions after treatment with 1 M KCl or 0.1 M Na_2_CO_3_. Under the high-salt (1 M KCl) and high-pH (0.1 M Na_2_CO_3_) conditions, peripheral membrane proteins and peripheral/luminal membrane proteins, respectively, can be released to the supernatant. In both cases, PlAMV replicase in the P30 fraction was detected in the nonsolubilized (P) fraction, while more than half the amount of BiP was solubilized by these treatments, similar to the observation from a previous study (Fig. 1D, lanes 3 to 6) (37). These results collectively suggested that PlAMV replicase is tightly associated with membranes and behaves as an integral membrane protein, similar to replication proteins of some other plant viruses (7).

### Tight membrane association was mediated by the MET domain of PlAMV replicase.

**FIG 2:**
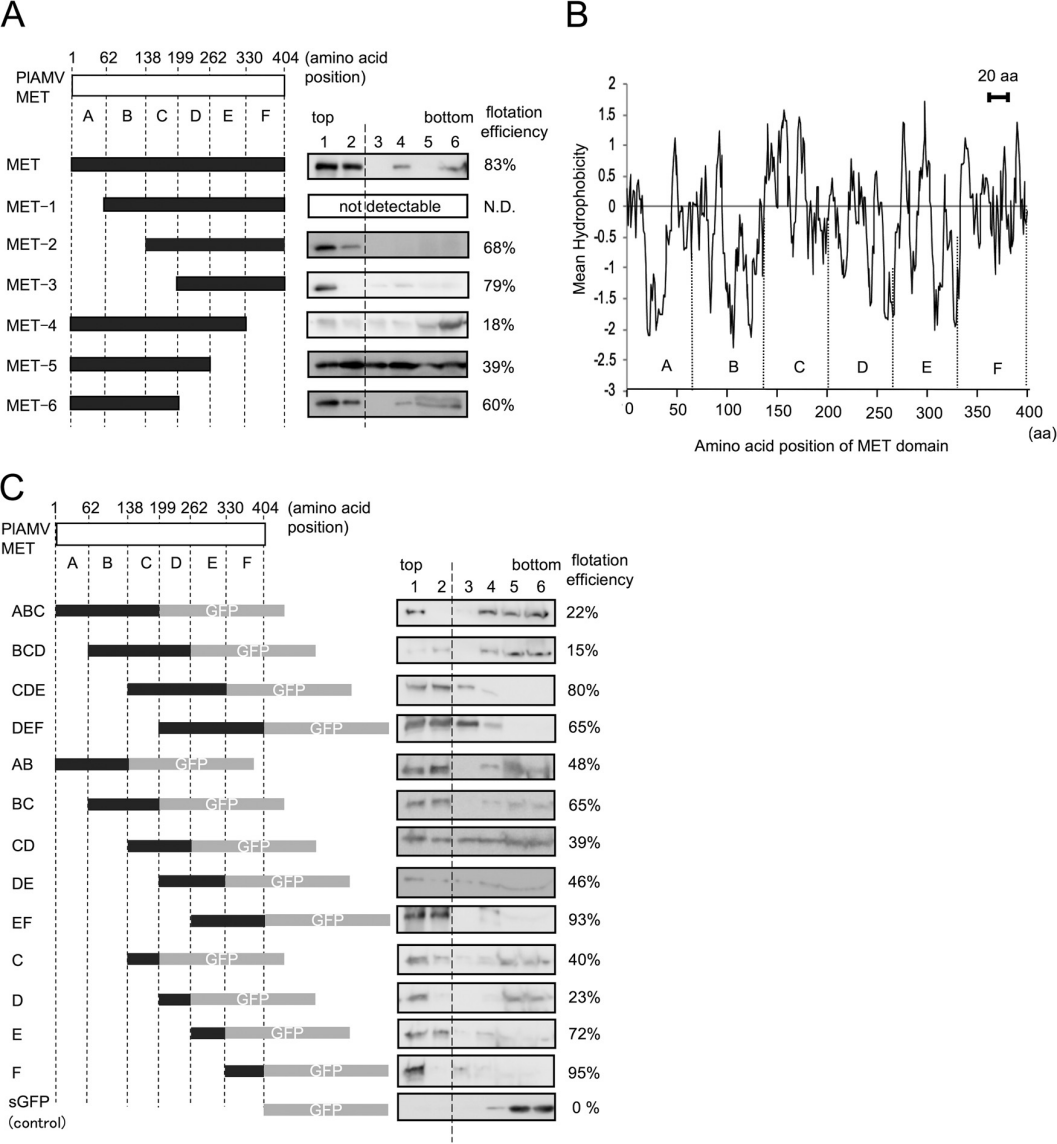
Identification of MET subdomains involved in tight membrane association. (A and C) Membrane flotation analyses were performed using transiently expressed MET domains tagged with c-*myc* epitope (A) and sGFP (C) at the C terminus and their deletion mutants. The deletion mutants of the MET domain were designed based on designated subdomains A through F. The numbers above the box represent the amino acid positions of the subdomain boundaries. Shown at left are schematic diagrams of the deletion mutants of the MET domain and at right membrane flotation profiles after immunoblot analysis. The c-*myc* epitope and sGFP tags were detected with anti-c-*myc* and anti-GFP antibodies, respectively. In panel C, unfused sGFP was used as a negative control. Flotation efficiencies were calculated as the percentage of detected proteins in the top two fractions of the gradient (delimited by dashed line) in the total detected proteins. MET-1 was not detected (A). (B) Hydrophobicity plot of the MET domain of PlAMV replicase. Hydrophobicity was calculated in the ExPASy webserver (http://web.expasy.org/protscale/) with the algorithm by Kyte and Doolittle, and mean hydrophobicity is displayed with a window size of 9 amino acids. A similar result was obtained with other algorithms. Locations of subdomains A to F of MET domain are shown above the *x* axis in the graph. A horizontal line on the top of the plot indicates the length of hydrophobicity needed to represent 20 consecutive amino acids necessary for a transmembrane domain.

To determine which functional domains of the replicase mediated membrane association, protein extracts were prepared from N. benthamiana leaves transiently expressing a single domain (MET, HEL, or POL) or a combination of adjacent domains (MET-HEL or HEL-POL), each fused with c-*myc* tag at their C terminus and then separated into supernatant (S30) and membrane (P30) fractions. Immunoblot analysis showed that MET, MET-HEL, and HEL-POL fractionated almost exclusively in the P30 fraction, while HEL and POL were present in both P30 and S30 fractions (Fig. 1E). These results indicated that the three predicted domains may be involved in membrane association by PlAMV replicase in mechanistically different manners. Nevertheless, MET was likely to have a tighter membrane association property than HEL and POL, based on its nearly exclusive fractionation into the P30 fraction. Additionally, the fractionation of MET into P30 was confirmed to result from membrane association and not from protein aggregation through the membrane flotation analysis (Fig. 2A). Based on these results, the MET domain was selected for further analysis to search for crucial amino acid sequences that govern its membrane association property.

### Identification of MET subdomains involved in tight membrane association.

Our results suggested that the MET domain should contain a transmembrane or hydrophobic region or regions that mediate membrane association. However, the hydrophobicity profile of the MET domain of PlAMV replicase (amino acids 1 to 404) indicated no hydrophobic amino acid regions that would be long enough to span the membranes (Fig. 2B). Hence, to identify membrane association regions, we generated deletion mutants of the MET domain on the basis of six subdomains (subdomains A to F) that each consisted of approximately 60 to 80 amino acids (Fig. 2A). These MET deletion mutants were transiently expressed in leaves of N. benthamiana by agroinfiltration, followed by protein extraction, membrane flotation, and immunoblot analysis. All MET deletion mutants except MET-1 were expressed at detectable levels (Fig. 2A), suggesting that the deletion of the N-terminal subdomain A might cause severe protein destabilization. The high flotation efficiencies of MET-2 (68%) and MET-3 (79%), which were similar to that of the full-length MET domain (83%), indicated that the C-terminal portion of the MET domain might be involved in its tight membrane association. This conclusion was supported by findings that the deletion of the subdomains in the C-terminal half resulted in the lower flotation efficiencies of MET-4 (18%), MET-5 (39%), and MET-6 (60%). In particular, the deletion of subdomain F alone substantially decreased the membrane association of the MET domain, indicating that subdomain F may be a key region to mediate tight membrane association of the MET domain.

To examine the contribution of MET subdomains to membrane association in more detail, we carried out transient expression assays with a series of single or combined subdomains that were fused with synthetic green fluorescent protein (sGFP) at their C termini. The membrane flotation and immunoblot analyses showed that the fusion proteins with either or both of the E and F subdomains (CDE, DEF, EF, E, and F) exhibited high flotation efficiencies (65 to 95%), except for the case of DE (46%), while sGFP used as a negative control did not float to the top two fractions (0%) (Fig. 2C). In addition, our results revealed that the fusion protein with the F subdomain alone showed the highest flotation efficiency (95%) (Fig. 2C), demonstrating that the F subdomain is sufficient to enable GFP to associate tightly with membranes. Regarding the E subdomain, these results indicated that it may, alone or in combination with other subdomains (e.g., subdomain C), have a role in enhancing the membrane association of the MET domain. In contrast, the other fusions showed the low to moderate flotation efficiencies (15% to 48%), except for the case of BC (65%) (Fig. 2C), suggesting that subdomains A to D have limited effects on membrane association (Fig. 2C).

### Structural analysis of the amphipathic helix in the membrane association subdomain F of the MET domain.

**FIG 3:**
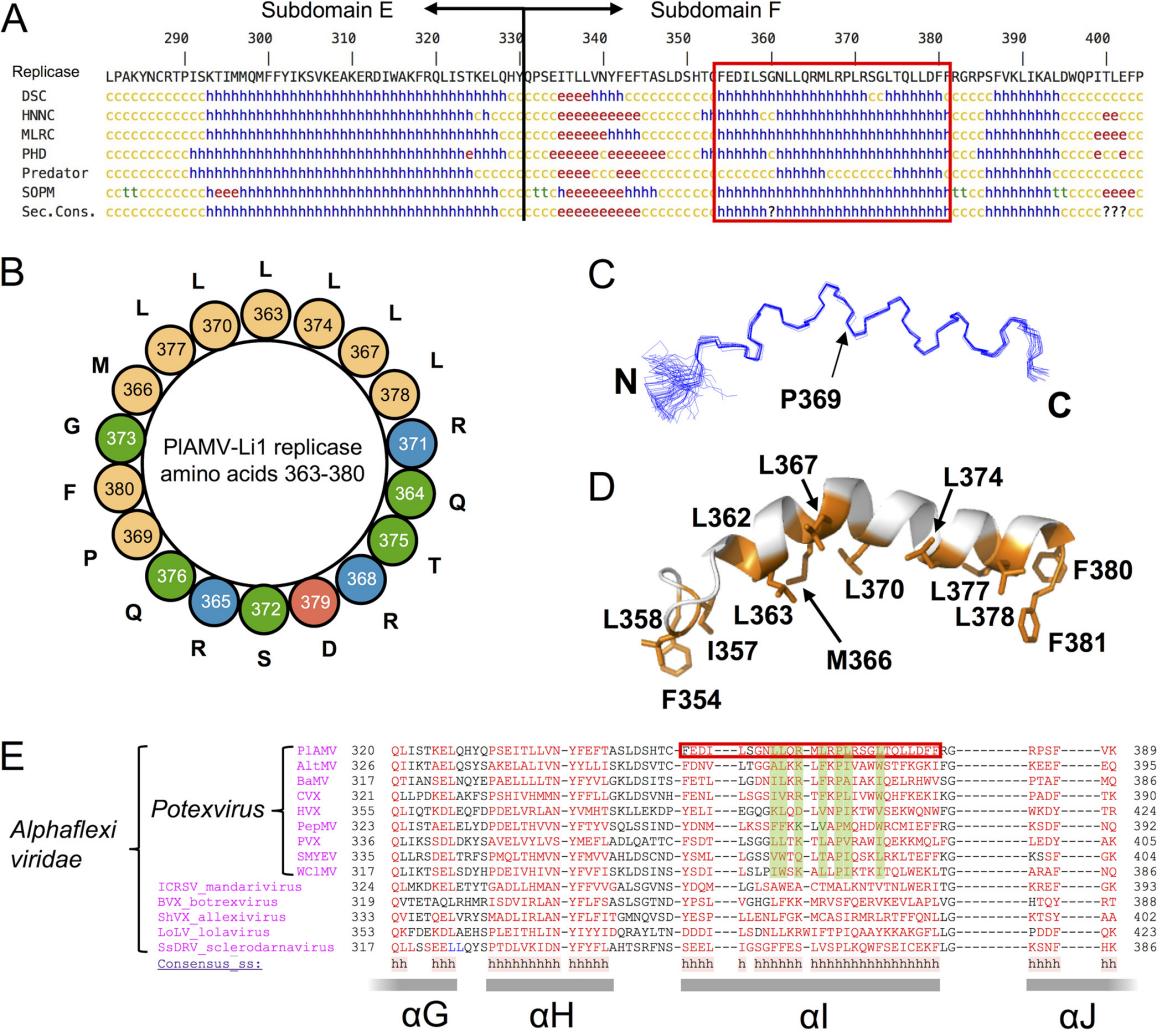
*In silico* prediction and NMR-based structure determination of the amphipathic α-helix in the MET domain. (A) Secondary structure prediction of the C-terminal region (part of subdomain E and the whole subdomain F) of the MET domain of PlAMV replicase using several algorithms, including DSC, HNNC, MLRC, PHD, Predator, and SOPM in the NPS@ website (82). Colored letters in the figure represent helical (h; blue), extended (e; red), turn (t; green), and coil (c; yellow). The consensus of the results of all algorithms is shown in the bottom line (Sec. Cons.). The segment enclosed by the red box indicates amino acid residues 354 to 381 of PlAMV replicase, which was used for structure determination by NMR. (B) Helical wheel projection of amino acid residues 363 to 380 of PlAMV replicase predicted using the HeliQuest program (47). Each color represents nonpolar (yellow), polar or uncharged (green), acidic (red), and basic (blue) amino acid residues. (C) Ensemble of 20 structures of peptide amino acids 354 to 381 analyzed by NMR. The N and C termini are indicated as N and C, respectively. P369 represents the proline residue where the helix is kinked. (D) Ribbon representation of the NMR structure. Nonpolar and hydrophobic residues (Ile, Leu, Met, and Phe) are shown in orange. (E) Amino acid sequence alignments of the C-terminal region of the MET domain of several viruses in the family *Alphaflexiviridae*, including viruses in the genus *Potexvirus*, using PROMALS (45). Accession numbers of the viral sequences used in the alignment are shown in Table S1. The red box highlights an amphipathic α-helix of PlAMV replicase, whose structure was resolved by NMR. Green shading indicates amino acid residues whose chemical properties were conserved in potexviruses. Gray thick bars below the alignment represent consensus secondary structure for all aligned virus sequences, named according to reference 39.

The region encompassing subdomains E and F (amino acids 262 to 404) is located downstream of the core MET enzymatic domain (methyltransferase-glycosyltransferase; amino acids 59 to 225) (38) and has no significant long stretches of hydrophobic amino acids (Fig. 2B). Membrane association domains have been predicted in the corresponding regions of the replicase of other viruses of the alphavirus supergroup (39). Predictions of secondary structure of subdomains E and F by several algorithms revealed that each subdomain might form an α-helix (Fig. 3A). A helical wheel projection of the putative α-helices indicated that the amino acid sequences in subdomains E and, particularly, F had propensities to form amphipathic α-helices with a stretch of hydrophobic amino acid residues on one side and hydrophilic and polar residues on the other side (Fig. 3B).

**FIG 4:**
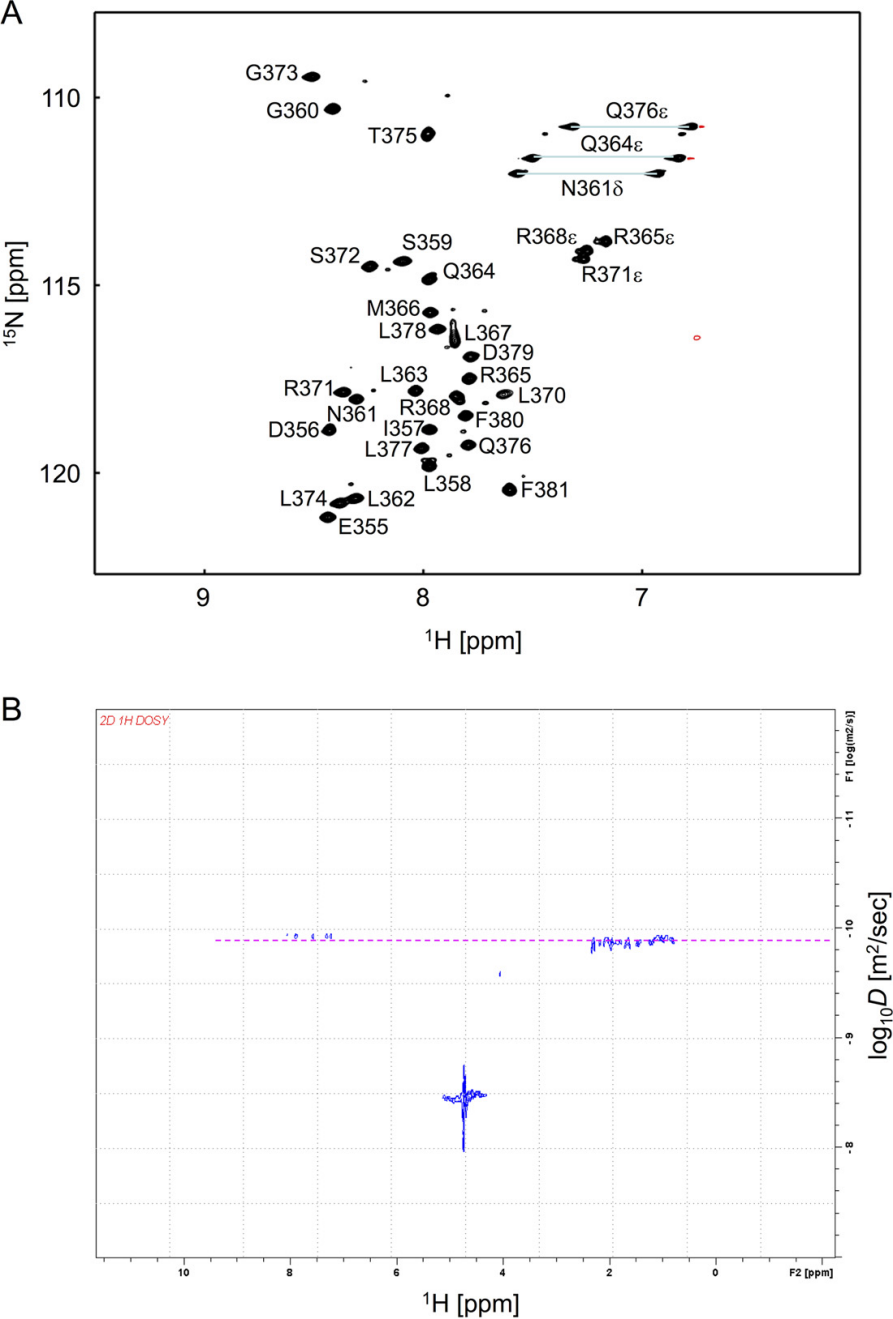
NMR structure of amino acids 354 to 381 of PlAMV replicase. (A) ^1^H and ^15^N heteronuclear single quantum correlation (HSQC) spectrum of the amino acid 354-to-381 peptides with an SDS micelle. The chemical shift assignments are labeled in the figure. (B) ^1^H DOSY spectrum of the 28-amino-acid peptide. The dashed line in magenta indicates the diffusion constant of the peptide, which is close to the theoretical value of an SDS micelle containing the peptide.

To confirm whether amino acids 354 to 381 of PlAMV replicase actually fold into an amphipathic α-helix, we synthesized a peptide of 28 amino acids by the cell-free protein expression system (40, 41) and analyzed its structure in the presence of SDS micelles by NMR. The chemical shift assignments for the almost all atoms except Phe 354 were achieved by using a series of the conventional two-dimensional (2D) and 3D NMR spectra (Fig. 4A). The NMR structural statistics of the selected 20 structures of the peptide (Table 1 and Fig. 3C) showed that the obtained three-dimensional structures are well converged, with a low root mean square deviation (RMSD) for the backbone atoms of 0.50 ± 0.23 Å, without distance or dihedral angle violations. Consistent with the helical wheel projection (Fig. 3B), a close examination of the α-helix revealed its amphipathic property with both hydrophobic and hydrophilic sides (Fig. 3D). Since the hydrophobic side of the helix is assumed to be closely associated with the lipid bilayer of intracellular membranes, the helix may be positioned in parallel to the plane of the membrane. In addition, a kink at Pro 369 with an angle of about 36.7° in this helix is another notable feature that has not been reported for any known membrane-associated amphipathic α-helices of virus proteins (11, 15, 17, 42, 43).

**TABLE 1:** Summary of conformational constraints and statistics of the final 20 structures of the synthesized peptide (Phe 354 to Phe 381) with an SDS micelle

Parameter	Result for parameter
NOE upper distance restraints	
Intraresidual (|*i* - *j*| = 0)	169
Medium range (1 ≤ |*i* − *j*| ≤ 4)	448
Long range (|*i* – *j*| > 4)	5
Total	622
Dihedral angle restraints (φ and ψ)	30
CYANA target function value (Å^2^)	0.05 ± 0.002
Mean AMBER energy (kcal/mol)	−1,319.24 ± 4.41
No. of violations	
Distance violations (>0.30 Å)	0
Dihedral angle violations (>5.0°)	0
Ramachandran plot (%)^*a*^	
Residues in most favored regions	90.6
Residues in additional allowed regions	9.4
Residues in generously allowed regions	0.0
Residues in disallowed regions	0.0
RMSD from averaged coordinates (Å)^*a*^	
Backbone atoms	0.50 ± 0.23
Heavy atoms	1.05 ± 0.25

a For residues Asn 361 to Phe 380.

Additionally, to ascertain whether the major population of this peptide interacts with the SDS micelles, we performed a diffusion-ordered NMR spectroscopy (DOSY) experiment. The DOSY spectra showed that the diffusion coefficient of the peptide was 1.26 × 10^−10^ [log_10_(−9.9)] m^2^/s (Fig. 4B). This value corresponded to a molecular weight of about 16,000 Da. Taking into account the molecular weights of the peptide (about 3,300 Da) and an SDS micelle (over 14,000 Da) (44), this result indicated almost all peptide molecules were attached to the SDS micelle in relationship of 1 or, at most, 2 peptides to 1 micelle.

### A membrane-associated amphipathic α-helix is conserved in the replicase of many related virus species.

To examine whether the amphipathic α-helix found in PlAMV replicase is commonly conserved in replicases of related plant RNA viruses, we performed a PROMALS alignment that is based on the predicted secondary structure of multiple amino acid sequences (45). Alignment analysis of replicase sequences from 14 virus species, including PlAMV in the family *Alphaflexiviridae*, revealed that the amphipathic α-helix sequence in PlAMV replicase was located in a conserved α-helix region named αI (39) (Fig. 3E). We further investigated whether the predicted α-helix structures in the αI region could be amphipathic by using the Heliquest program (46, 47). As a result, all analyzed viruses in the families *Alphaflexiviridae*, *Gammaflexiviridae*, and *Tymoviridae*, but not those in the *Betaflexiviridae*, were predicted to possess an amphipathic α-helix in their αI region (see Table S1 in the supplemental material). Interestingly, despite the limited conservation and apparent variability of the amino acid sequences of the αI region, chemical properties of some amino acid residues were highly conserved in the potexvirus sequences analyzed (Fig. 3E). In particular, proline residues corresponding to Pro 369 of PlAMV replicase were completely conserved. Furthermore, hydrophobic amino acids (i.e., isoleucine, leucine, tryptophan, phenylalanine, and valine) predominated at positions corresponding to those of Leu 362, Leu 363, Leu 367, Leu 370, and Leu 374 of PlAMV replicase. We also noticed that basic polar amino acids were highly conserved at the position corresponding to that of Arg 365 of PlAMV replicase. These results implied that the conserved chemical properties of the amino acid residues in the αI region or the amphipathic α-helix structure might be important for membrane association of potexvirus replicases as well as virus replication.

### Mutations in the amphipathic helix abolish PlAMV replication in protoplasts.

**FIG 5:**
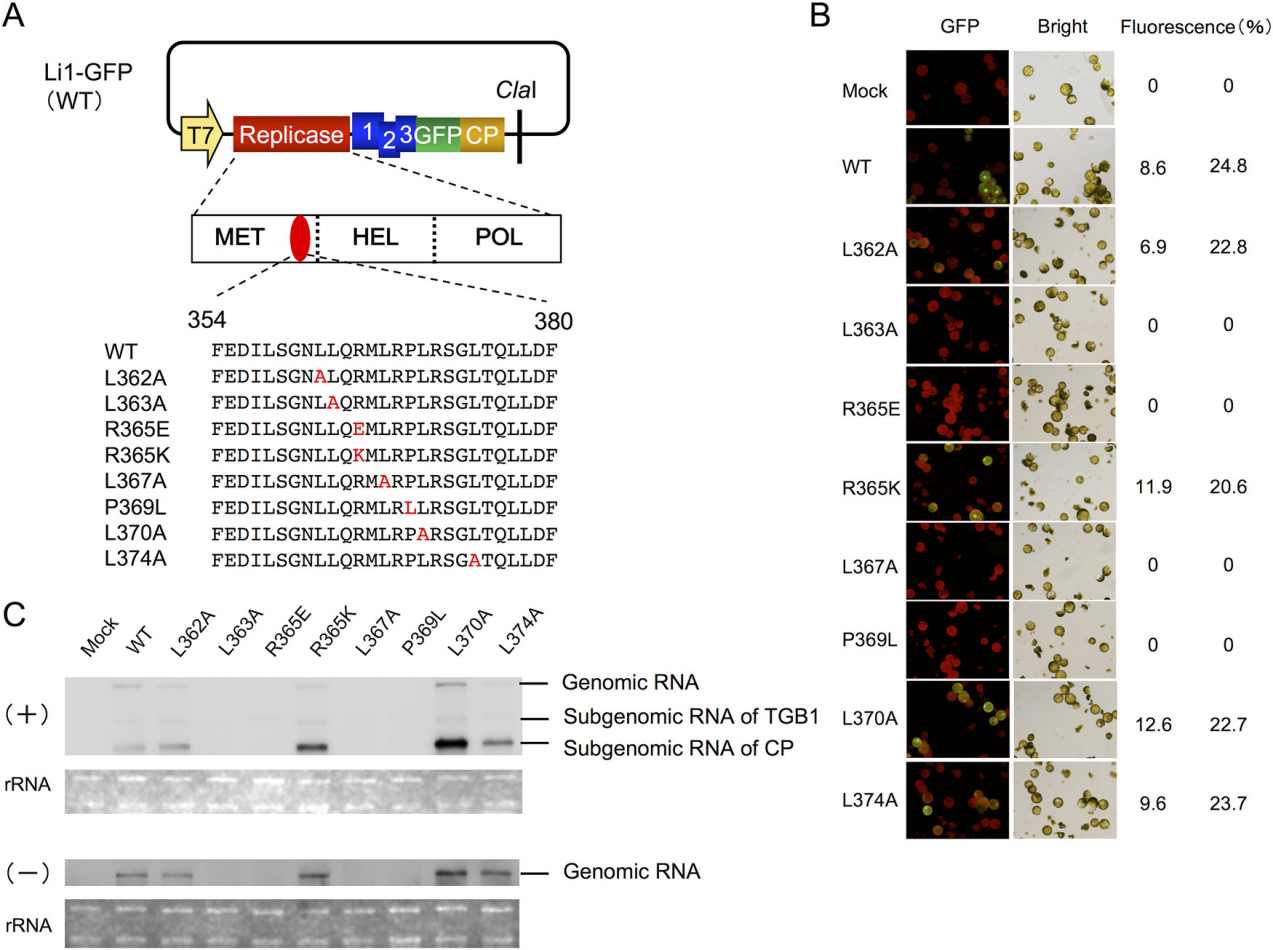
Protoplast infection analysis of mutant viruses. (A) Schematic diagram of the GFP expression vector Li1-GFP and its mutants used for inoculation. Colored rectangles indicate open reading frames in the virus sequence. The yellow arrow indicates T7 polymerase promoter sequence. Amino acid residues 354 to 380 of the MET domain of replicase containing the membrane-associated amphipathic α-helix are shown for the wild-type Li1-GFP and its mutant viruses. Amino acid substitutions are shown in red. (B) Fluorescent images of protoplasts 24 h post-polyethylene glycol-mediated inoculation with viral genomic RNA of wild-type (WT) Li1-GFP and the mutant viruses containing single-amino-acid substitutions in the amphipathic α-helix region. Cells exhibiting GFP fluorescence were counted and scored as the percentage of the total number of cells exhibiting green fluorescence in two fields over the total number of cells in the two fields. Percentages at the right for each mutant represent those obtained in two independent experiments. The total numbers of cells observed per mutant per experiment ranged from 44 to 220. (C) At 24 h postinoculation, total RNA was extracted from protoplasts, and 500 ng of total RNA per virus was subjected to Northern blot analysis with DIG-labeled RNA probes that could specifically detect plus-strand (+) and minus-strand (−) RNAs of PlAMV. rRNA stained by ethidium bromide is shown as a loading control.

To elucidate the role of the amphipathic α-helix in virus replication, we generated eight replicase mutants of PlAMV and tested their replication abilities in protoplasts. For this experiment, we introduced mutations for hydrophobic leucines (L362A, L363A, L367A, L370A, and L374A), the polar arginine (R365E and R365K), and the kink-associated proline (P369L) in the amphipathic α-helix (Fig. 5A). Inoculation of N. benthamiana protoplasts with *in vitro* transcripts of Li1-sGFP, representing the GFP-tagged parental virus, and its mutants showed that cells infected with Li1-sGFP or one of the L362A, R365K, L370A, or L374A mutants exhibited strong GFP fluorescence at 24 h postinoculation (hpi), while inoculation with each of the other four mutants (L363A, R365E, L367A, and P369L) resulted in no GFP fluorescence (Fig. 5B). Northern blotting of total RNAs extracted from inoculated protoplasts further demonstrated that Li1-sGFP and the L362A, R365K, L370A, and L374A mutants were able to amplify positive-strand genomic and subgenomic RNAs as well as negative-strand genomic RNAs to detectable levels (Fig. 5C). In contrast, neither positive-strand nor negative-strand viral RNAs were detected in protoplasts inoculated with the L363A, R365E, L367A, and P369L mutants (Fig. 5C). These results suggested that specific amino acid residues in the amphipathic α-helix have a critical role in the early phase in PlAMV replication.

### A role of the amphipathic helix in ER localization of the MET domain.

**FIG 6:**
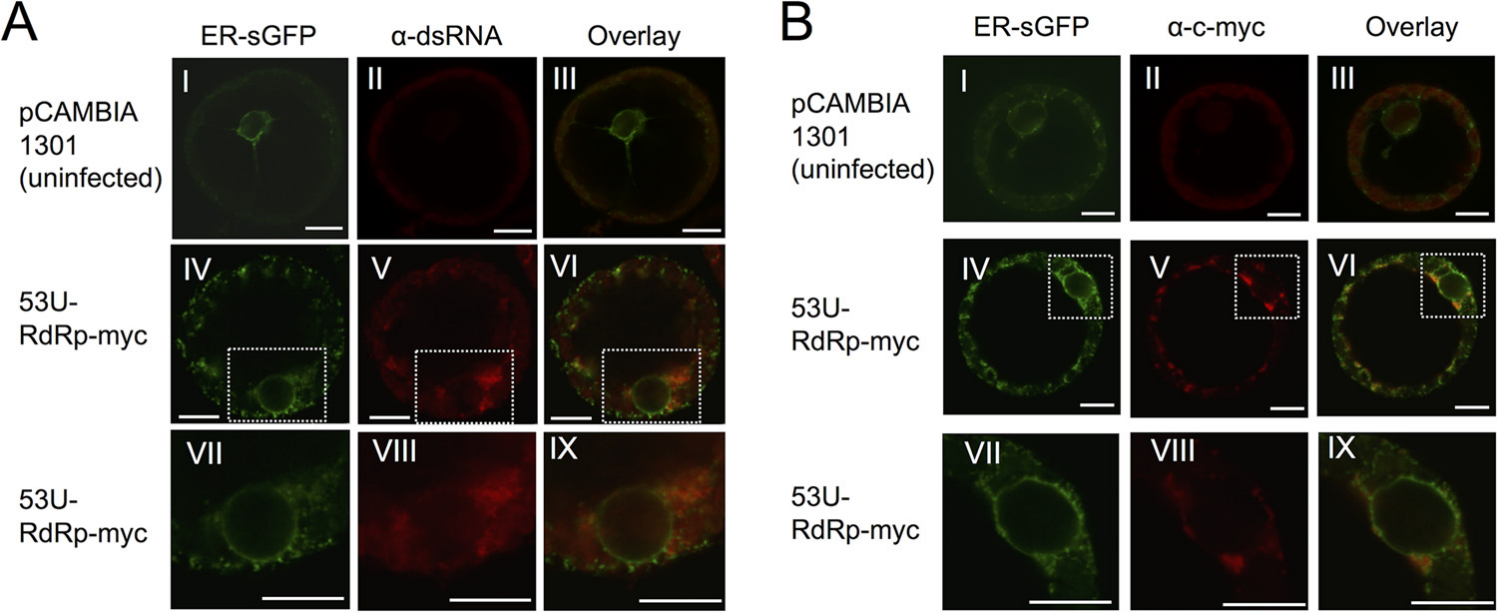
Fluorescent immunostaining of replicase and dsRNA replication intermediate of PlAMV in the context of infection. Protoplasts were prepared 2 days post-agroinfiltration with the PlAMV replicon, treated with anti-dsRNA (A) or anti-c-*myc* (B) primary antibody, and subsequently treated with Alexa Fluor 546-conjugated goat anti-rabbit antibody (red signal). Protoplasts were prepared from N. benthamiana leaves infiltrated with the control vector pCAMBIA1301 (subpanels I to III) or 53U-RdRp-1m-myc (subpanels IV to IX) as well as with ER-sGFP (green signal). Images were taken by confocal laser-scanning microscopy. Subpanels VII to IX are magnified images of the boxed area of subpanels IV to VI, respectively, showing the perinuclear region. Size bars, 10 μm.

Considering that the amphipathic α-helix of PlAMV replicase might be involved in both the ER membrane association of the MET domain (Fig. 2) and virus replication (Fig. 5), we hypothesized the possibility that the amphipathic α-helix might also play an important role in targeting the replicase to ER membranes for VRC formation. To explore this possibility, we first determined how PlAMV replicase was associated with ER membranes in plant cells by immunocytochemistry and CLSM observation. Viral double-stranded RNA (dsRNA), which is an intermediate of viral replication (48), was also detected to compare its location with that of the replicase during viral replication. We transiently coexpressed the PlAMV replicon 53U-RdRp-myc, together with an ER marker, ER-sGFP (49), by agroinfiltration into N. benthamiana leaves, isolated protoplasts from the infiltrated regions at 24 hpi, and detected the replicase and viral dsRNA with anti-c-*myc* and anti-dsRNA antibodies, respectively. 53U-RdRp-myc-infected cells showed that strong signals of PlAMV replicase and dsRNA were localized in close association with ER-sGFP at both peripheral and perinuclear regions (Fig. 6A and B). Notably, we found that aggregates of the ER marker were formed at many perinuclear sites where PlAMV replicase and dsRNA accumulated (Fig. 6A and B). These ER aggregates were indicative of the formation of VRC and also reminiscent of “X-bodies” observed in perinuclear regions of potexvirus-infected cells (24, 25).

**FIG 7:**
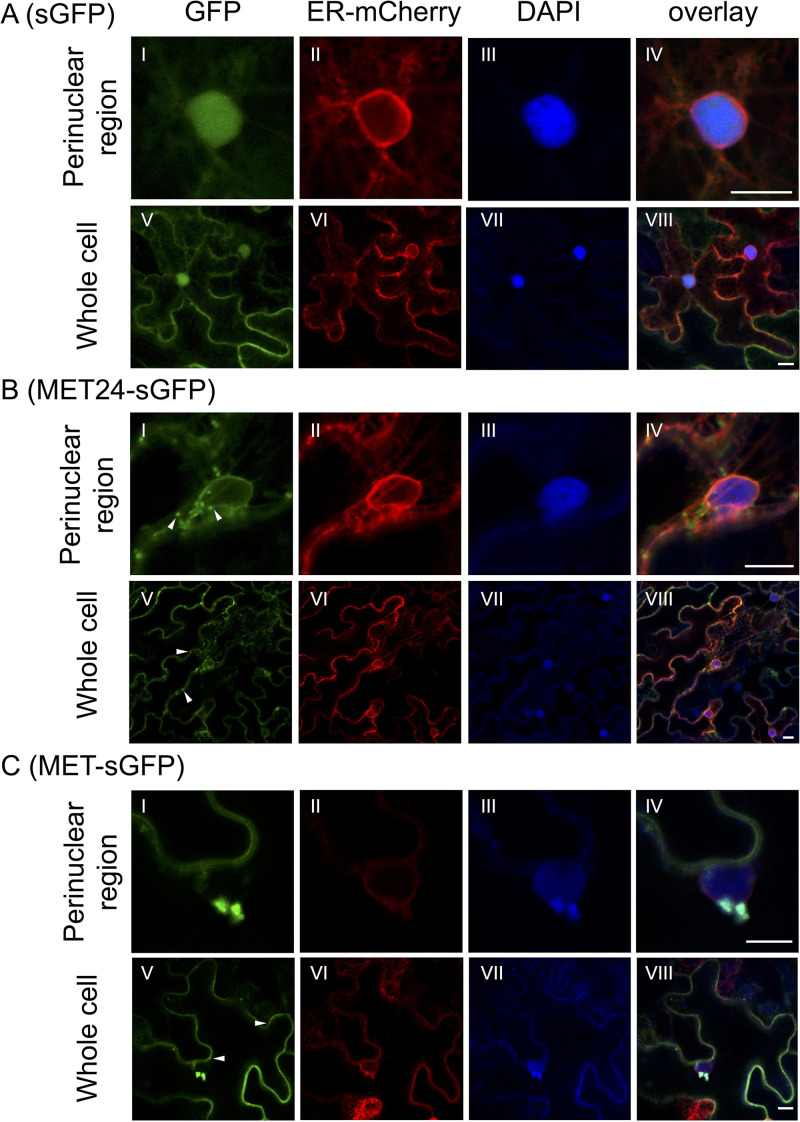
Confocal laser scanning microscopy (CLSM) of 24-amino-acid amphipathic α-helix domain (MET24) or full-length MET domain (MET) fused with sGFP. Shown are representative CLSM images of the perinuclear region (subpanels I to IV) and the whole cell (subpanels V to VIII) of N. benthamiana epidermal cells expressing nonfused sGFP (A), MET24 fused with sGFP (B), or MET fused with sGFP (C), together with ER-mCherry (subpanels II and VI). Cell nuclei were stained by DAPI in the same samples (subpanels III and VII). Subcellular localization was observed at 24 h after infiltration. Each image presents a single section of the cell. Arrowheads indicate examples of peripheral small punctate structures. Size bars, 10 μm.

Next, to investigate the ER membrane association property of the amphipathic α-helix in the replicase, we observed the subcellular location of 24 amino acid residues in the helical sequence (MET24: L358 to F381) fused with sGFP at its C terminus (MET24-sGFP). We performed agroinfiltration into N. benthamiana to transiently coexpress MET24-sGFP or a control nonfused sGFP together with an ER marker, ER-mCherry. Our CLSM observation at 24 hpi showed that fluorescent signals of MET24-sGFP were associated with the ER network, whereas those of sGFP were detected in the nucleus and the cytoplasm (Fig. 7A and B). Specifically, MET24-sGFP was often found as small punctate structures of about 0.5 μm in diameter along the ER networks in the perinuclear (Fig. 7B, subpanels I to IV) and peripheral (Fig. 7B, subpanels V to VIII) regions. These observations indicated that the MET24 sequence was enough for targeting to and associating with ER membranes.

**FIG 8:**
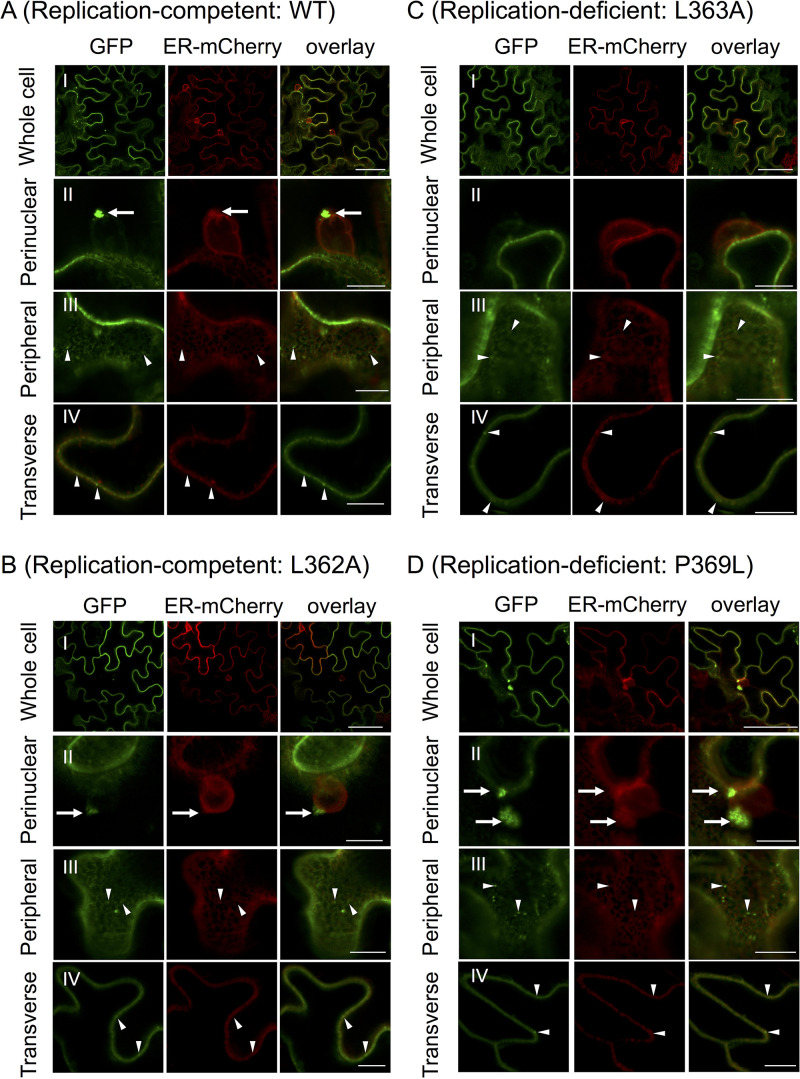
Confocal laser scanning microscopy (CLSM) of MET domain mutants fused with sGFP. Shown are representative CLSM images of the whole cell (subpanel I), perinuclear (subpanel II) and peripheral (subpanel III) regions, and transverse section of the cell edge (panel IV) of N. benthamiana epidermal cells expressing the wild type (WT) (A) and single-amino acid mutants (B, L362A; C, L363A, and D, P369L) of the MET domain of PlAMV replicase fused with sGFP. Replication-competent mutants (R365K, L370A, and L374A) and replication-deficient mutants (R365E and L367A) displayed similar subcellular localization patterns to L362A (B) and L363A (C), respectively (Fig. 9). Subcellular localization of these GFP fusion proteins coexpressed with ER-mCherry, an ER marker, was observed at 24 h after agroinfiltration. Each image presents a single section of the cell. Arrows and arrowheads indicate perinuclear large complexes and peripheral small punctate structures, respectively. Size bars, 50 μm (the whole cell in subpanel I) and 10 μm (the other images in subpanels II to IV).

In addition to MET24-sGFP, we also analyzed the subcellular location of a whole MET domain (amino acids 1 to 404) fused with sGFP at its C terminus (MET-sGFP). At 24 hpi, not only small punctate structures (about 0.5 μm in diameter) but also a large complex (more than 2.5 μm in diameter) of MET-sGFP were observed along ER networks in the perinuclear and peripheral regions of infiltrated cells (Fig. 7C and 8A and Table 2). The large complexes in the perinuclear region were colocalized with the dense ER-mCherry structures, suggesting that ER membranes were remodeled by the transient expression of MET-sGFP (Fig. 7C, subpanels I to IV), similar to the case of infection with 53U-RdRp-myc (Fig. 6). The large complexes of MET-sGFP were basically static (see Movie S1 in the supplemental material), except for some cases in which large complexes moved along the ER networks (see Movie S2 in the supplemental material). Upon closer observation, large complexes seemed to be composed of many small punctate structures (Fig. 7C, subpanel I); however, as we observed no small punctate structures trafficking to or from a large complex during our observation period, the relationship between the two structures remains unclear. These results indicated that regions of the MET domain outside the MET24 fragment might contain an additional functional region or regions that cause the formation of large intracellular complexes in the perinuclear region.

**TABLE 2:** Observation of large complexes formed by MET-GFP and its single-amino-acid mutants

Expressed protein	Replication in protoplasts^*a*^	No. (%) of cells^*b*^:			
		With LCs			Without LCs
		>2.5 μm	Associated with nucleus	Not associated with nucleus	
MET (WT)-GFP	+	22 (73.3)	18 (60.0)	4 (13.3)	8 (26.7)
L362A-GFP	+	18 (60.0)	8 (26.7)	10 (33.3)	12 (40.0)
L363A-GFP	−	3 (10.0)	2 (6.7)	1 (3.3)	27 (90.0)
R365E-GFP	−	2 (6.7)	0 (0.0)	2 (6.7)	28 (93.3)
R365K-GFP	+	25 (83.3)	12 (40.0)	13 (43.3)	5 (16.7)
L367A-GFP	−	0 (0.0)	0 (0.0)	0 (0.0)	30 (100)
P369L-GFP	−	27 (90.0)	18 (60.0)	9 (30.0)	3 (10.0)
L370A-GFP	+	12 (40.0)	4 (13.3)	8 (26.7)	18 (60.0)
L374A-GFP	+	13 (43.3)	3 (10.0)	10 (33.3)	17 (56.7)

a Summary of the results shown in Fig. 5. +, Li1-GFP or its single-amino-acid mutants replicated in protoplasts; –, mutant failed to replicate.

b The total number of cells examined for each expressed protein was 30. LCs, large complexes.

**FIG 9:**
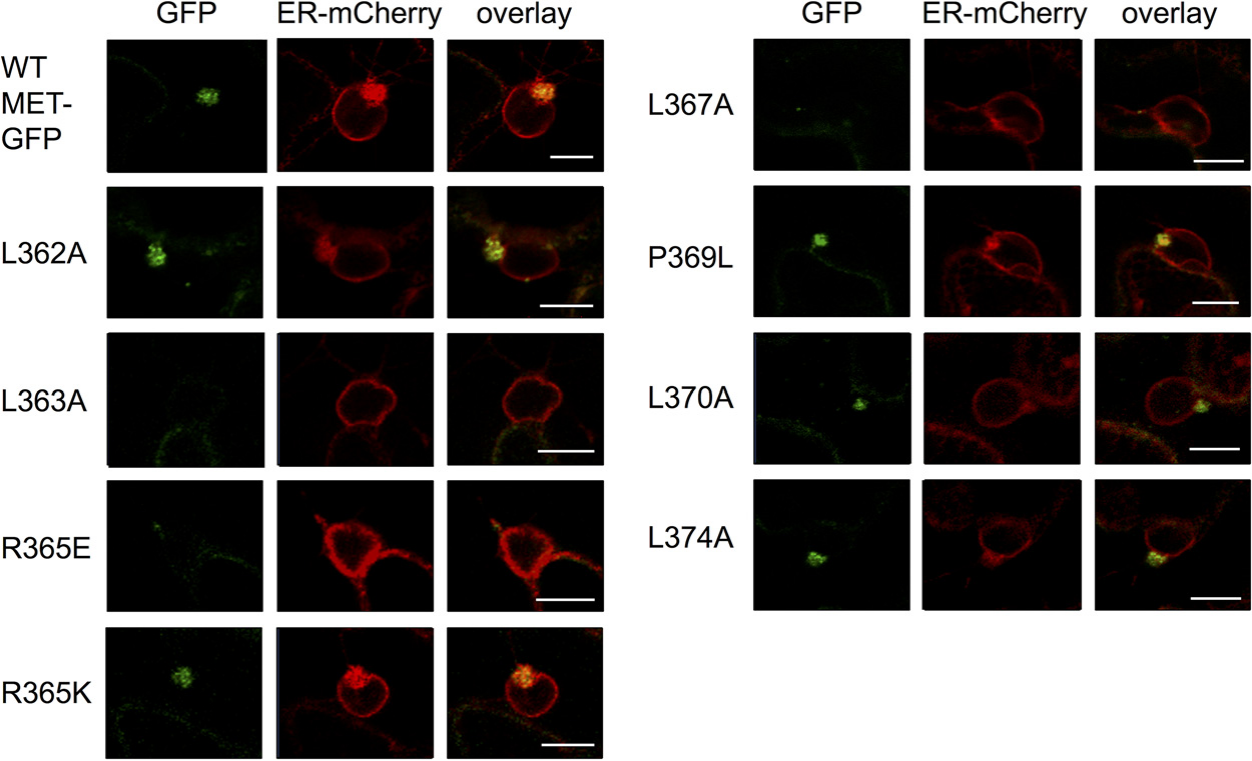
Confocal laser scanning microscopy (CLSM) images of N. benthamiana leaves expressing single-amino-acid mutants of MET-sGFP and ER-mCherry, an ER marker. Cells were observed at 24 h postinfiltration. MET-sGFP constructs containing L363A, R365K, P369L, L370A and L374A mutations continued to express green fluorescence in large perinuclear complexes, similar to those observed for MET-sGFP not containing any amino acid substitutions (WT MET-GFP). Size bars, 10 μm.

We examined the subcellular location of mutants of MET24-sGFP and MET-sGFP, each of which contained one of the eight amino acid substitutions shown in Fig. 5A. Subcellular localization patterns of MET24-sGFP mutants were indistinguishable from that of the wild-type MET24-sGFP shown in Fig. 7B. On the other hand, MET-sGFP mutants showed different abilities to form large intracellular complexes. MET-sGFP mutants with any of the four mutations that had no influence on virus replication in protoplasts (L362A, R365K, L370A, and L374A) induced large complexes in the perinuclear region similar to those induced by unsubstituted MET-sGFP (Fig. 8A and B, subpanel II; Table 2; and Fig. 9). In contrast, three of the four mutations that disrupted virus replication in protoplasts (L363A, R365E, and L367A) abolished or decreased the formation of large complexes in the perinuclear region (Fig. 8C, subpanel II; Table 2; and Fig. 9). Interestingly though, the MET domain containing the replication-defective mutation (P369L) did retain the ability to form large complexes in the perinuclear region (Fig. 8D, subpanel II; Table 2; and Fig. 9). All MET-sGFP mutants, however, retained the ability to form small punctate structures in the peripheral region (Fig. 8, subpanels III and IV). These results suggest that the property of MET domain to form small punctate structures or a large complex might be necessary, but not sufficient, for PlAMV replication. Additionally, these results suggest that the amphipathic α-helix of PlAMV replicase required other regions in the MET domain to form large complexes similar to those formed by the virus replicase (Fig. 6B) during VRC formation.

### Replicase with P369L mutation in the amphipathic helix retains the ability to form the high-molecular-weight complex *in vitro*.

**FIG 10:**
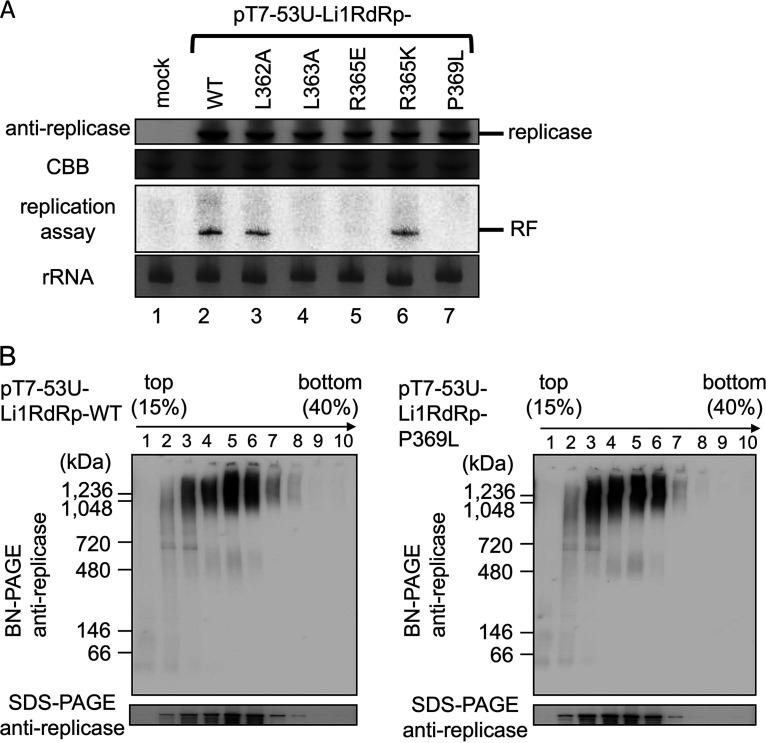
*In vitro* translation/replication assays and detection of the high-molecular-weight replicase complexes of PlAMV replicons. (A) *In vitro* replication assay of WT PlAMV replicon and its variants containing single-amino-acid substitutions in the amphipathic α-helix. Twenty-five microliters of BYL reaction mixture was added with water (indicated as mock, lane 1), 1 μg RNA transcribed from pT7-53U-Li1RdRp-WT (lane 2), or 1 μg RNA of each mutant (lane 3 to 7) and subjected to immunoblot analysis using anti-PlAMV replicase antibody and to an *in vitro* replication assay. Coomassie brilliant blue (CBB)-stained proteins and methylene blue-stained rRNAs are presented as loading controls of immunoblot analysis and the *in vitro* replication assay, respectively. The positions of PlAMV replicase (approximately 156 kDa) and replicative-form RNA (RF) are shown on the right. (B) Sucrose gradient sedimentation to detect the high-molecular-weight replicase complex of PlAMV. Ten micrograms of RNA transcribed from pT7-53U-Li1RdRp-WT (left) or -P369L (right) was incubated in 200 μl of BYLS30 reaction mixture for 1 h at 25°C and mixed with 5 μl of 10 mM puromycin, and 180 μl of mixtures was fractionated by continuous 15 to 40% sucrose gradient sedimentation. Ten fractions were subjected to BN-PAGE and SDS-PAGE, followed by immunoblot analysis using anti-PlAMV replicase antibody.

Findings in our recent study using an *in vitro* PVX translation/replication system have suggested that a potexvirus replicase is part of the high-molecular-weight premature VRC considered to assemble before membrane association and to be crucial for viral replication (35). This finding prompted us to analyze whether the mutations within the amphipathic α-helix of the full-length PlAMV replicase affected its *in vitro* replication capacity and the formation of the replicase complex. *In vitro*-transcribed wild-type (WT) PlAMV replicon RNA (clone pT7-53U-Li1RdRp-WT) and its mutant RNAs that contained L362A, L363A, R365E, R365K, or P369L substitutions were incubated in BYL, the vacuole- and nucleus-free extract of tobacco BY-2 protoplasts (50). At 1 h of incubation, after addition of puromycin to terminate translation, similar levels of the replicase were detected by immunoblot analysis, indicating that none of the mutations changed translation efficiency (Fig. 10A). To examine the ability of each mutant to replicate *in vitro*, ribonucleoside triphosphates and [α-^32^P]CTP were added to the translation mixtures and incubated for another 1 h. Replicative-form (RF) RNA was detected by autoradiography in replication reaction mixture samples containing WT, L362A, and R365K, but not L363A, R365E, and P369L, transcripts (Fig. 10A). Consistent with the result from the protoplast experiment (Fig. 5), this result indicated that certain amino acid residues in the amphipathic α-helix have a critical role in the early phase in PlAMV replication.

Then, to address whether the replication defect caused by a mutation in the amphipathic α-helix was associated with the incomplete formation of a replication-associated complex, we examined the effect of P369L on the formation of high-molecular-weight replicase complexes *in vitro*, because this substitution maintained the ability to associate with ER and induce large complexes (Fig. 8). WT or P369L PlAMV replicon RNAs were incubated in BYL supernatant (BYLS30) for 1 h for translation of the replicase and formation of premature VRC (35), followed by continuous sucrose density gradient centrifugation and fractionation. Blue native PAGE (BN-PAGE) and subsequent anti-PlAMV-replicase immunoblot analysis showed that both WT and P369L PlAMV replicons produced the high-molecular-weight replicase complex (>1,000 kDa) in fractions 3 to 7 (Fig. 10B), indicating that the P369L mutation does not appear to affect the formation of the high-molecular-weight replicase complex. Additional studies indicated that the formation of the high-molecular-weight replicase complex was better correlated with appearance of small punctate structures than with the large complexes since the other mutant replicases that could form small punctate structures but not form large complexes or replicate *in planta* also could form the high-molecular-weight replicase complexes *in vitro* (T. Yoshida, unpublished data).

## DISCUSSION

The association of the replicase of plant RNA viruses with cellular membranes of host plant cells, without exception to date, is required for virus replication. In previous studies of several animal and plant viruses, amphipathic α-helix structures within their replicases were important for associating with and remodeling cellular membranes for virus replication (10). Thus, we assumed that the replicase of PlAMV might have one or more membrane-associated domains containing amphipathic α-helix structures. In the present study, using membrane flotation assays, we determined that PlAMV replicase is associated with intracellular membranes, including the ER, and that its three functional domains (MET, HEL, and POL) have different membrane association properties. We further showed that the MET domain has no predicted transmembrane domains but contains an α-helix amphipathic region with a proline-induced kink and forms intracellular complexes localized to the perinuclear region where the viral replicase and dsRNA reside (Fig. 4 and Fig. 6 to 9). The importance of this amphipathic α-helix region was demonstrated by protoplast infection assay using mutants altered in conserved amino acid residues within the region. Moreover, based on the correlation between complex formation and the ability to replicate in protoplasts, with the exception of a kink-associated proline mutant, it is possible that MET-based formation of large perinuclear complexes is required for successful replication by PlAMV, but it is definitely not sufficient for that activity (Fig. 5 and 8).

Our structural analyses predicted that the αI region, located within subdomain F of the MET domain, forms an amphipathic α-helix in the replicase of several potexviruses and other related viruses in the order *Tymovirales*. This region is within a broader region, referred to as the “Iceberg region,” which is defined by the conserved secondary structure content of α-helices and β-strands but not the amino acid sequence (39). Although not in the Iceberg region, a similar amphipathic α-helix was also identified in the MET core domain of the replicase of grapevine rupestris stem pitting-associated virus, a member of the related *Betaflexiviridae* family, and was responsible for membrane association (51). In addition, our NMR-based analysis using the 28-amino-acid fragment in the MET domain of PlAMV replicase provided the first experimental evidence of the proline-kinked amphipathic α-helix structure in a potexvirus replicase. This detailed structural determination made it easy to select and examine amino acid residues, conserved for chemical qualities, within the α-helix for their influence on membrane association by region and virus replication. Through our experiment substituting amino acids in the amphipathic α-helix of the MET domain of the PlAMV replicase, we successfully identified amino acid residues with important roles in intracellular localization of the MET domain and/or virus replication. However, these amino acids within the helical structure may not be the only targets to modify in future functional studies. Given that the physical structure of the region and chemical characteristics of individual amino acids may be more important than the specific amino acid for the function of the amphipathic α-helix, the investigation of nonconserved amino acids within the amphipathic α-helices of PlAMV and related virus species for function should be pursued.

Specific findings within our predictive function analyses based on PlAMV amphipathic α-helix structure analysis demonstrated that substitution of conserved hydrophobic and kink-associated amino acid residues in the region (L363A, R365E, L367A, and P369L) prevented PlAMV from replicating in protoplasts (Fig. 5). It is possible that the hydrophobicity of the leucine residues (Leu 363 and Leu 367), the positive charge of the arginine residue (Arg 365), and the kink by the proline residue (Pro 369) in the amphipathic α-helix structure are essential for maintaining a specific structure required for RNA replication by PlAMV replicase. A previous study of the 1a replication protein of BMV showed that simultaneous mutations of multiple leucine residues and a polar residue in its amphipathic α-helix also abolished virus replication (11). In addition, mutations of leucine residues of the hydrophobic face of the amphipathic α-helix in 140K/98K replication proteins encoded by turnip yellow mosaic virus (TYMV) led to loss of replication (14). Our finding that not only positive-strand, but also negative-strand formation is inhibited by alteration of an amphipathic α-helix region indicates that this inhibition in replication occurs at the initial phases of virus replication.

Regarding the relationship of intracellular complex formation by viral components with replicase membrane association, recent confocal and electron microscopic analyses indicate that the replicase of PVX was localized to ER-derived, TGBp2/p3-containing granular vesicles at 24 h postinoculation in protoplasts (23, 24). Granular vesicles visualized by GFP-fused TGBp3 appeared as early as 12 hpi in protoplasts and were observed at the leading edge of infection in leaves (23). Moreover, the TGBp2 of PVX was concomitantly shown to form large perinuclear bodies (called X-bodies), which contain both replicase and a dsRNA replication intermediate (52). It was suggested that the granular and perinuclear structures may be involved in the early stage of replication of potexviruses (52). Similar to the findings for PVX, in our subcellular localization observations during ectopic expression of MET-GFP and MET24-GFP, we determined that MET-GFP, which encompasses all MET subdomains (amino acids 1 to 404), formed ER-associated, small punctate structures and large intracellular complexes and that MET24-GFP localized to ER-associated small punctate structures. Interestingly, none of the eight single-amino-acid substitutions in the amphipathic α-helix altered the formation of ER-associated small punctate structures by either MET-GFP or MET24-GFP. This includes the four amino acids that were found to be important for virus replication. Our results are consistent with previous findings that mutations in the amphipathic α-helix of 1a protein of BMV and p27 of red clover necrotic mosaic virus (RCNMV) can inhibit virus replication but not affect membrane association of the proteins (11, 12). These findings indicate that replicase association with membranes and formation of small punctate structures are not sufficient for virus replication. The function of this region beyond these two roles is now a topic of importance for future work.

Additionally, it is interesting that the proline substitution in the amphipathic α-helix domain resulted in MET-GFP that could produce both small punctate structures and large intracellular complexes (Fig. 8). Our close observation of these large complexes suggests that they result from a gathering of small punctate structures of MET-GFP (Fig. 7C, subpanel I). In addition, such perinuclear complexes are reminiscent of ER membrane aggregates and X-bodies that were observed around the nucleus in cells infected by PlAMV (in this study) and other potexviruses (24, 25, 52). On the assumption that the appearance of these ER membrane aggregates as well as X-bodies may reflect the formation of VRCs, our results suggest that the proline substitution in the virus still allows the formation of these intracellular complexes, but this is not sufficient for virus negative-strand synthesis. If these intracellular complexes indeed reflect VRC formation, then formation of this complex, assumed normal at the resolution of our imaging, is not sufficient for this mutant virus to accumulate, and again, the amphipathic α-helix region has additional function(s) beyond the large complex formation necessary for PlAMV replication. This idea is also supported by our *in vitro* translation/replication assays using wild-type and P369L PlAMV replicons. BN-PAGE and immunoblot analysis revealed that the P369L mutant could form high-molecular-weight replicase complexes (>1,000 kDa), which are reminiscent of those proposed to be essential for potexviral replication in our recent study (35). Although it remains to be determined whether there is identity between the structure of the high-molecular-weight complexes shown by BN-PAGE and the large complexes observed by CLSM, we conclude here that the P369L mutant may be able to form VRCs that are structurally similar to those formed by wild-type PlAMV.

The finding that replication-deficient P369L can form high-molecular-weight replicase complexes is different from that reported for RCNMV, which showed that mutations into the amphipathic helix of its accessory replication protein, p27, abolished both viral replication and the formation of the VRC (12). In contrast, an example of a lack of linkage between formation of membrane-associated complex and virus replication, similar to PlAMV, is known for p33 of tomato bushy stunt virus (TBSV). The N-terminal region of TBSV p33 is not required for membrane localization of the protein but is required for replication (53). Ubiquitination of two lysine residues in this N-terminal region of p33 is shown to be required for recruitment of Vps23p ESCRT-I protein to the peroxisomal membrane for replication (54, 55). P369L replication complex may not be able to interact properly with as yet undetermined host factors needed to exert its replication activity. Alternatively, P369 may be required for the interaction with sterols and phospholipids. Indeed, at the N-terminal region of TBSV p33, tyrosine residue 42 was found to be important for direct binding to sterols, which is essential for viral replication (56). Another possible role of P369 may be enriching sterols and phospholipids or promoting their redistribution to the replication complex. TBSV p33 induces the formation of membrane contact sites with the help of co-opted host factors, which contribute to the alternation of lipid composition of VRC (57–59). A recent study has shown that enzymes involved in lipid biosynthesis and lipid modifications are transported to the replication complex of TBSV by the host retromer complex (60). The P369L replicase complex may fail to establish an optimized microenvironment appropriate for replication through recruitment of host factors. Future research should examine whether lipid composition and host factors involved in lipid biosynthesis play a role in PlAMV replication and whether the MET domain can serve as the master regulator of PlAMV replication, like p33 of TBSV (61).

In summary, here we have shown that the replicase of PlAMV has a novel, potexvirus-specific proline-kinked amphipathic α-helix in the MET domain and that this amphipathic α-helix plays an essential role in virus replication. The importance of a proline-kinked amphipathic α-helix was corroborated by a previous study on a movement protein (MP) encoded by prunus necrotic ringspot virus, which showed that a replacement of a proline residue in the middle of a hydrophobic region of MP prevented cell-to-cell movement of the virus (62). We also determined that the formation of intracellular complexes by the MET domain can be unlinked from virus replication, as observed for the proline residue substitution within the MET domain of PlAMV, where intracellular complexes formed but virus did not replicate. In animal viruses, the amphipathic α-helix has been a promising target for pharmacological inhibition of virus replication (63, 64). Further investigation of functional amphipathic α-helix structures conserved among plant viruses in different genera will not only provide new insights into molecular mechanisms of virus replication but also help us develop extensively effective antivirus chemicals.

## MATERIALS AND METHODS

### Construction of plasmids expressing replicase domains and mutant viruses.

pER8-RdRp-myc, representing an estradiol-inducible expression plasmid containing the full-length replicase with a c-*myc* tag at its C terminus, plasmids expressing each domain of replicase of PlAMV Li1 isolate (GenBank accession no. AB360790) with the C-terminal c-*myc* tag (p53U-MET, -MET-HEL, -HEL, -HEL-POL, and -POL), and 53U-RdRp-myc, a PlAMV replicon containing the replicase ORF and the flanking untranslated regions, were described previously (33). A binary plasmid vector for expressing the GFP-tagged ER marker (ABRC stock no. CD3-955) (49) and mCherry-tagged ER marker (pBIC/ER-mCherry) (65) was used for colocalization analyses.

Plasmids for transient expression of a truncated MET domain (used in Fig. 2A) were constructed as follows. A truncated MET domain sequence was made using p53U-MET as a template and with primers designed to amplify subdomains A to F (Table 3). To amplify MET-1, −2, −3, −4, −5, and −6 sequences, primer sets MET-62A-F and Li-MD-R, MET-138E-F and Li-MD-R, MET-200E-F and Li-MD-R, Li-MD-F and MET-330Y-R, Li-MD-F and MET-262T-R, and Li-MD-F and MET-199S-R, respectively, were used. All amplified fragments were digested with MfeI (corresponding to amino acid residues 73 to 74 of p53U-MET just downstream of the translational start codon of p53U-HEL) and SpeI (located before the c-*myc* tag) and cloned into p53U-MET (for MET-1, −2, and −3) or p53U-HEL (for MET-4, −5, and −6) digested with the same restriction enzyme sites.

**TABLE 3:** Names and sequences of primers used in this study

Primer name	Primer sequence (5′→3′)
Li-MD-F	GGCTCTAGAGTCGACATGTCAAACGTTCGGAACGT
MET-62A-F	AATCAATTGCCATCACCCCCCACACCCA
MET-137L-R	TTTAACTAGTCAGGCGTTTGATGATCGTGT
MET-138E-F	AATCAATTGAAAACATCCCCACCAGCATAG
MET-199S-R	ATTACTAGTGGAGTAGGTCAGTGTGTAGA
MET-200E-F	AATCAATTGAGGACGGGTTCATGTACAT
MET-262T-R	ATTACTAGTAGGGGTGAGGTAGTTGCCC
MET-263E-F	AATCAATTGAAAGACGTACCTTCACCACC
MET-330Y-R	ATTACTAGTGTAATGTTGGAGCTCCTTGG
MET-331Q-F	AATCAATTGAACAGCCATCCGAGATCACCC
MET358L-F	GGAATTCTTTCCGGGAATCTCCTACA
MET381F-R	TTTAACTAGTGAAGAAGTCCAGCAGTTGTGT
MET358L-363A-F	GGAATTCTTTCCGGGAATCTCGCACAGCGAATGCTCCGGCCGCT
Li-MD-R	TTTAACTAGTGGGAAATTCCAAGGTGATGG
T7-Li1-1F	GTAATACGACTCACTATAGAAAACAAACCTACACAAACCA
PlAMV-3508R	TCGCAGTTTATCATGATGTTGTTGG
polyT-ClaI^*a*^	ATCGATTTTTTTTTTTTTTTTTTTTTTTTTTTTTTTV
Li-L362A-F	AAGACATACTTTCCGGGAATGCTCTACAGCGAATGCTCCGGCC
Li-L362A-R	GGCCGGAGCATTCGCTGTAGAGCATTCCCGGAAAGTATGTCTT
Li-L363A-F	ACATACTTTCCGGGAATCTCGCACAGCGAATGCTCCGGCCGCT
Li-L363A-R	AGCGGCCGGAGCATTCGCTGTGCGAGATTCCCGGAAAGTATGT
Li-R365E-F	TTTCCGGGAATCTCCTACAGGAAATGCTCCGGCCGCTGAGGTC
Li-R365E-R	GACCTCAGCGGCCGGAGCATTTCCTGTAGGAGATTCCCGGAAA
Li-R365K-F	TTTCCGGGAATCTCCTACAGAAAATGCTCCGGCCGCTGAGGTC
Li-R365K-R	GACCTCAGCGGCCGGAGCATTTTCTGTAGGAGATTCCCGGAAA
Li-L367A-F	GGAATCTCCTACAGCGAATGGCACGGCCGCTGAGGTCAGGCCT
Li-L367A-R	AGGCCTGACCTCAGCGGCCGTGCCATTCGCTGTAGGAGATTCC
Li-P369L-F	TCCTACAGCGAATGCTCCGGCTACTGAGGTCAGGCCTCACACA
Li-P369L-R	TGTGTGAGGCCTGACCTCAGTAGCCGGAGCATTCGCTGTAGGA
Li-L370A-F	TACAGCGAATGCTCCGGCCGGCAAGGTCAGGCCTCACACAACT
Li-L370A-R	AGTTGTGTGAGGCCTGACCTTGCCGGCCGGAGCATTCGCTGTA
Li-L374A-F	TCCGGCCGCTGAGGTCAGGCGCTACACAACTGCTGGACTTCTT
Li-L374A-R	AAGAAGTCCAGCAGTTGTGTAGCGCCTGACCTCAGCGGCCGGA
vec20nt-Li1F	TTGTAATACGACTCACTATAGAAAACAAACCTACACAAACCAAACG
C-26A-ClaI-vec11nt_R	GCATGCATCGATTTTTTTTTTTTTTTTTTTTTTTTTTG
T7_invR	TATAGTGAGTCGTATTACAATTCACTGG
7A-ClaI-vec11nt_invF	AAAAAAATCGATGCATGCAAGCTTTTGTTC

a V represents any nucleotide except T.

In order to construct plasmid vectors for transient expression of MET subdomains fused with GFP at their C terminus (used in Fig. 2C), a cloning vector, pCAMBIA1301.1-sGFP, was first constructed by modifying pCAMBIA1301. The original multicloning site was deleted from this construct, and its GUS gene, which was expressed under the control of cauliflower mosaic virus 35S promoter, was replaced with the sGFP gene flanked by new multicloning sites. Each partial MET segment (ABC, BCD, CDE, DEF, AB, BC, CD, DE, EF, C, D, E, and F) was amplified by PCR as described above with primers listed in Table 3. All amplified segments except AB and ABC were digested with MfeI and SpeI and cloned into pCAMBIA1301.1-sGFP digested with EcoRI and SpeI, which were both located upstream of the sGFP sequence, to yield constructs expressing MET segments fused at their C terminus with sGFP. To generate AB-sGFP and ABC-sGFP, amplified fragments and pCAMBIA1301.1-sGFP were digested with SalI and SpeI, and fragments were cloned into the vector as described for the other fragments. Full-length MET-sGFP was produced by the same strategy described above using primer pair Li-MD-F and Li-MD-R. For construction of MET24-sGFP containing the 24 amino acids that constitute amphipathic α-helix fused with C-terminal sGFP, the primer pair MET358L-F and MET381F-R were used to amplify 72 nucleotides (nt) representing the 24 amino acids. Full-length MET and MET24 were cloned into the EcoRI and SpeI sites of pCAMBIA1301.1-sGFP.

To generate single-amino-acid-mutated infectious cDNA clones of PlAMV expressing GFP, the plasmid T7-Li1-sGFP, from which infectious genomic RNA of PlAMV expressing sGFP is transcribed (66), was used. T7-Li1-sGFP was constructed from the agroinfection-compatible binary vector pLi1-CPNsGFP-mtfCP (66, 67). Using T7-Li1-sGFP as a template, specific point mutations in the MET24 region were introduced by PCR with eight pairs of overlapping forward and reverse primers (Li-L362A-F to Li-L374A-R) containing the desired nucleotides to modify particular amino acid residues (Table 3). An approximately 1,100-bp fragment was amplified with PlAMV-1F and each reverse (R) primer containing the codon for the substituted amino acid, and an approximately 2,400-bp fragment was amplified with each forward (F) primer containing the codon for the substituted amino acid and PlAMV-3508R. These two overlapping fragments were fused through joint PCR with primers 1F and 3508R. The resulting PCR products were digested with MfeI (nt 304 of the PlAMV genome sequence) and BglII (nt 3212 of the PlAMV genome sequence) and cloned into T7-Li1-GFP digested with the same restriction enzyme sites.

Single-amino-acid substitutions within MET-sGFP and MET24-sGFP were introduced by PCR amplification of the corresponding full-length MET or MET24 regions using the above-described eight mutants of T7-Li1-GFP as the templates, with Li-MD-F and Li-MD-R primers for MET and MET358L-F and MET381F-R for MET24 (Table 3), and then cloning fragments into the EcoRI- and SpeI-digested pCAMBIA1301.1-sGFP. One exception was the L363A mutant of MET24-sGFP. For this construct, the forward primer MET358L-363A-F (Table 3) was used instead.

For *in vitro* translation and replication assays, pT7-53U-Li1RdRp-WT was constructed first. A fragment of the Li1 genome that contains the entire replicase ORF and the 5′ and 3′ untranslated regions was amplified using p53U-RdRp1 (33) with the primer set vec20nt-Li1F and C-26A-ClaI-vec11nt_R (Table 3). In parallel, a fragment containing the vector background of pT7-53U-UK3RdRp-WT (35) was amplified with the primer set T7_invR and 7A-ClaI-vec11nt_invF (Table 3). Both fragments were gel purified and combined with Gibson Assembly master mix (New England Biolabs, Ipswich, MA, USA) to make pT7-53U-Li1RdRp-WT. Single-amino-acid substitution was introduced by amplifying an approximately 3.5-kbp fragment using the T7-Lil-sGFP mutant constructs described above as a template, with primer set T7-Li1-1F and PlAMV-3508R, and by cloning these fragments into EcoRI- and MfeI-digested pT7-53U-Li1RdRp-WT.

For constructions of all of the plasmids described above, PCR amplifications were performed using the high-fidelity DNA polymerase KOD-Plus (Toyobo, Osaka, Japan), and introduced substitutions were verified by Sanger sequencing.

### Peptide synthesis for NMR spectroscopy.

The ^13^C, ^15^N-labeled polypeptide corresponding to amino acid residues 354 to 381 of PlAMV replicase, with its N-terminal hexahistidine and a small ubiquitin-modifying protein (SUMO) tags, was synthesized in a cell-free protein expression system (68). The synthesized peptide was purified by nickel affinity purification and anion-exchange column chromatography, followed by cleavage with SUMO protease (69). The tag-cleaved fraction was applied to another nickel affinity column, and its flowthrough fraction was pooled. The purified protein was dissolved in 0.1% trifluoroacetic acid and applied to a reverse-phase column chromatography (Jupiter 10-μm, C_18_, 300-Å liquid chromatography [LC] column, 250 by 10 mm [Phenomenex]) for fractionation. The fractionated sample was freeze-dried and then was dissolved in 20 mM d_3_-NaOAc (pH 5.6) containing 100 mM deuterated SDS and 10% D_2_O for further analysis.

### NMR spectroscopy and structure determination.

NMR spectra were acquired at 310K with a Bruker 600 MHz spectrometer equipped with a cryoprobe (Bruker Biospin, Karisruhe, Germany). Chemical shift assignments were obtained using ^1^H,^15^N-heteronuclear single quantum correlation (HSQC),^1^H,^13^C-HSQC, HNCO, HN(CA)CO, HN(CO)CA, HNCA, CBCA(CO)NH, HNCACB, HBHA(CO)NH, C(CO)NH, HC(C)H-total correlation spectroscopy (TOCSY), (H)CCH-TOCSY, and HC(C)H-correlation spectroscopy (COSY) for the aromatic region (70, 71).^15^N-edited nuclear Overhauser effect spectroscopy (NOESY) and ^13^C-edited NOESY with 200-ms mixing times were used to calculate the distance restraints. All spectra were processed with NMRPipe (72). Visualization of the NMR spectra and chemical shift assignments were performed using the NMRView and KUJIRA programs (73, 74). The DOSY spectrum was processed and analyzed with Topspin (Bruker Biospin, Rheinstetten, Germany).

Structure calculation with automated NOE cross-peak assignments and torsion angle dynamics were carried out using the program CYANA (75). In this calculation, dihedral angle restraints derived from the program TALOS were included with the error margins ± 30° (76). The final structure calculations with CYANA were started from 100 conformers with random torsion angle values. The 20 conformers with the lowest final CYANA target function values were further refined with the AMBER14 program, using an Amber ff14SB force field and a generalized Born model, as described previously (77). A summary of the statistics is described in Table 1. The resultant NMR structure was visualized using MolMol (https://archive.org/details/tucows_9805_MOLMOL) and PyMOL (http://www.pymol.org/).

### Plant growth conditions and agroinfiltration.

Nicotiana benthamiana plants were grown in a growth chamber under 16 h light/8 h dark and 25°C. All binary plasmids were transformed into Agrobacterium tumefaciens strain EHA105 for transient gene expression (agroinfiltration). Agroinfiltration was performed essentially as described previously (78), with a final optical density at 600 nm (OD_600_) of 1.0 for *Agrobacterium* in agroinfiltration buffer (10 mM MES [morpholineethanesulfonic acid], 10 mM MgCl_2_, and 150 μM acetosyringone [pH 5.6]). For coexpression of multiple genes, equal volumes of each Agrobacterium tumefaciens suspension were mixed before infiltration. For estradiol-mediated transient expression of replicase, agrobacterium containing pER8-RdRp-myc was coexpressed with agrobacterium containing RNA silencing suppressor p19 of tomato bushy stunt virus to increase the replicase expression level. In this case, estradiol treatment was initiated 2.5 days after agroinfiltration with pER8-RdRp-myc and p19. At 2.5 days after the estradiol treatment, protein was extracted for further analysis.

### Subcellular fractionation and biochemical treatment of proteins, and immunoblot analysis.

Total protein extraction and fractionation into supernatant (S30) and membrane-containing pellet (P30) fractions were carried out as described previously, using buffer A (50 mM Tris-HCl [pH 7.5], 15 mM MgCl_2_, 120 mM KCl, 0.1% β-mercaptoethanol, 20% glycerol, and one tablet of cOmplete Mini Protease Inhibitor Cocktail [Roche Diagnostics, Mannheim, Germany] per 10 ml of buffer A) (33). Biochemical treatment and membrane flotation analysis of the P30 fraction containing PlAMV replicase were performed essentially as described previously (79). For biochemical treatment, the P30 fraction was dissolved in 500 μl of 100 mM Na_2_CO_3_ (pH 10.5) or 1 M KCl per 1 g of a leaf sample and incubated at 4°C for 30 min. The resultant solution was centrifuged at 30,000 × *g* at 4°C for 30 min to obtain soluble (S) and pellet (P) fractions. In each chemical treatment, the final volumes of the S and P fractions were equalized before immunoblot analysis.

For membrane flotation analysis, the P30 fraction was dissolved in 2.75 ml of buffer A to 52% (wt/wt) sucrose in a final volume, 6.25 ml of 45% and 1 ml of 10% sucrose solutions were layered onto this, and the mixture was centrifuged at 120,000 × *g* at 4°C for 20 h. Six to eight fractions of equal volume were manually taken, and each fraction was concentrated by methanol/chloroform precipitation (80). The resulting pellet was dissolved in Laemmli sample buffer for SDS-PAGE analysis (81).

Proteins were separated by SDS-PAGE on a Tris-glycine-polyacrylamide gel and were transferred onto a polyvinylidene difluoride membrane. For electrophoresis of the full-length replicase of PlAMV, which has a molecular weight of approximately 156 kDa, 3 to 8% Tris-acetate NuPAGE gel or 4 to 12% Bis-Tris NuPAGE gel (Thermo Fisher Scientific, Waltham, MA, USA) was used. For BN-PAGE analysis, NativePAGE 3 to 12% Bis-Tris protein gel (Thermo Fisher Scientific) was used. Immunoblot analyses were performed using mouse monoclonal anti-c-Myc antibody (clone 4A6, 1:1,000; EMD Millipore, Darmstadt, Germany), mouse monoclonal anti-GFP antibody (1:1,000; catalog no. 11814460001, Roche), rabbit polyclonal anti-PlAMV-replicase antibody (1:2,000) (33), and/or rabbit polyclonal anti-luminal binding protein (BiP) antibody (at-95, 1:2,000; Santa Cruz Biotechnology, Santa Cruz, CA, USA). Signals were detected with the ImmunoStar LD chemiluminescent reagents (Wako Pure Chemical Industries, Osaka, Japan) or with the Clarity Western ECL enhanced chemiluminescence substrate (Bio-Rad Laboratories, Hercules, CA, USA).

### Secondary structure prediction and amino acid sequence analysis.

Secondary structure prediction for the MET domain of PlAMV replicase (GenBank accession no. BAG12123) was carried out at the NPS@ (Network Protein Sequence Analysis) website (https://npsa-prabi.ibcp.fr/) (82). Helical wheel projection was created at the HeliQuest website (http://heliquest.ipmc.cnrs.fr) (47). An amino acid sequence alignment of replicases from several tymoviruses was created with the PROMALS program, which makes use of secondary structure annotation and can visualize the conserved secondary structure with the alignment (45). Amphipathic α-helix structure was predicted with the Amphipaseek software at the NPS@ site and the HeliQuest software (46, 47). Amphipaseek was used with high-specificity and low-sensitivity conditions. For a helix predicted by Amphipaseek, HeliQuest was used with 18-residue windows to analyze the region surrounding it. The HeliQuest discrimination factor (*D*), which depends on mean hydrophobic moment (〈μ*H*〉) and net charge (*z*), is defined as *D *= 0.944 (〈μ*H*〉) + 0.33 (*z*). Whether an amphipathic α-helix was predicted or not was judged by the following criteria: if *D* is ≥1.34, an amphipathic α-helix is reliably predicted, and if 0.68 < *D* < 1.34, an amphipathic α-helix is “potentially” predicted.

### Protoplast preparation, transcription, and inoculation of infectious transcripts, and Northern blotting analysis.

T7-Li1-GFP and the virus clones containing the mutations within the MET24 region were digested with a ClaI site just downstream of the poly(A) sequence. Virus RNAs were transcribed from the linearized plasmids using the MEGAscript T7 transcription kit (Thermo Fisher Scientific) with an m^7^GpppG cap analog (catalog no. S1404; New England Biolabs). Prior to protoplast inoculation, the quality and integrity of RNA were confirmed after transcription by polyacrylamide gel electrophoresis and staining with SYBR gold nucleic acid gel stain (Thermo Fisher Scientific). Protoplast preparation, polyethylene glycol (PEG)-mediated transfection, and incubation were performed as described previously (66). After incubation, total RNA was extracted from protoplasts, and 500 ng of the total RNA was used for Northern blot analyses using the digoxigenin (DIG) system (Roche Diagnostics) following the manufacturer’s protocols. DIG-labeled RNA probes for virus RNA detection were prepared by *in vitro* transcription from a plasmid containing cDNA of the viral CP gene (for detection of positive-strand virus RNAs) (32) and cDNA of the replicase gene (for detection of negative-strand virus RNAs) (33).

### Immunostaining and confocal microscopy.

Immunostaining of N. benthamiana protoplasts prepared from agroinfiltrated plants was performed as described previously, using plants at 2 days post-agroinfiltration (83). The constructs used for agroinfiltration were ER-GFP (49), 53U-RdRp-Li1-myc (33), and a control vector, pCAMBIA1301. The primary antibodies used were anti-c-Myc antibody at 1:200 (clone 4A6; EMD Millipore) and anti-dsRNA antibody at 1:100 (clone J2; English and Scientific Consulting Bt., Szirak, Hungary). The secondary antibody was Alexa Fluor 546-conjugated goat anti-rabbit antibody at 1:1,000 (A11010; Thermo Fisher Scientific). DAPI (4′,6-diamidino-2-phenylindole) staining solution (10 μg/ml) was infiltrated into leaves with a needleless syringe 30 min before imaging. Imaging analyses with a confocal laser scanning microscope (LSM710NLO; Carl Zeiss, Jena, Germany) were performed as described previously (84). sGFP and mCherry were excited with an argon laser at 488 nm and a diode-pumped solid-state laser at 561 nm, and emitted light was captured in windows of 493 and 556 nm and between 573 and 621 nm, respectively. DAPI was excited with 405-nm light, and emitted light was captured in the range between 410 and 528 nm. All fluorescence images were acquired with sequential scanning, and image processing was performed with the Carl Zeiss ZEN 2012 software.

### *In vitro* translation/replication assays and sucrose gradient sedimentation.

BYL was prepared and *in vitro* translation/replication assays were performed as described previously (35). RNAs for *in vitro* translation/replication assays were transcribed using ClaI-linearized pT7-53U-Li1RdRp-WT or its mutants as a template. For this assay, transcription was performed using the AmpliCap-Max T7 High Yield Message Maker kit (Cellscript, Madison, WI, USA) as described previously. For the detection of high-molecular-weight replication complex, BYL supernatant after centrifugation at 30,000 × *g* was used instead of BYL. Sucrose gradient sedimentation was also carried out as described previously (35).

### Data availability.

The NMR chemical shifts in this study have been deposited in the Biological Magnetic Resonance Data Bank under accession no. 36361. The final coordinates were deposited in the Worldwide Protein Data Bank under accession no. 7CK5.
