# Supplementary material for: Identification of a Proline-Kinked Amphipathic α-Helix Downstream from the Methyltransferase Domain of a Potexvirus Replicase and Its Role in Virus Replication and Perinuclear Complex Formation
Source: J Virol. 2021 Sep 27;95(20):e01906-20. doi: 10.1128/JVI.01906-20 (PMC8475525; doi:10.1128/JVI.01906-20)
Supplement: Supplemental file 4 — Supplemental Movie Legends. Download JVI.01906-20-s0002.pdf, PDF file, 0.04 MB [file jvi.01906-20-s0002.pdf]

Movie S1. A relatively immobile large body in the peripheral region formed by MET-sGFP. Time-lapse CLSM images of *N. benthamiana* leaves expressing the full-length MET-sGFP and ER-mCherry by agroinfiltration at 24 hours post infiltration.

Movie S2. Rare movement of a large body formed by MET-sGFP along the ER network. Time-lapse CLSM images of *N. benthamiana* leaves expressing the full-length MET-sGFP and ER-mCherry by agroinfiltration at 24 hours post infiltration.
